# Limbic‐Predominant Cortical Magnetic Susceptibility Alterations Are Associated With Glycemic Dysregulation and Cognitive Performance in Type 2 Diabetes

**DOI:** 10.1002/cns.71181

**Published:** 2026-09-25

**Authors:** Kun Wang, Dan Hu, Jiahe Wang, Ziyu Diao, Yuna Chen, Xuan Huang, Die Shen, Jie An, Shijun Qiu, Gang Li

**Affiliations:** ^1^ The First Clinical Medical College, Guangzhou University of Chinese Medicine Guangzhou Guangdong China; ^2^ Department of Radiology and Biomedical Research Imaging Center University of North Carolina at Chapel Hill Chapel Hill North Carolina USA; ^3^ Department of Radiology The First Affiliated Hospital of Guangzhou University of Chinese Medicine Guangzhou Guangdong China; ^4^ Department of Imaging Diagnostics Nanfang Hospital, Southern Medical University Guangzhou Guangdong China; ^5^ Department of Radiology Jinling Hospital, Affiliated Hospital of Medical School, Nanjing University Nanjing Jiangsu China; ^6^ State Key Laboratory of Traditional Chinese Medicine Syndrome Guangzhou Guangdong China

**Keywords:** cerebral cortex, cognitive impairment, hyperglycemia, magnetic susceptibility, quantitative susceptibility mapping, Type 2 diabetes mellitus

## Abstract

**Aims:**

To characterize the spatial pattern of cortical magnetic susceptibility alterations in Type 2 diabetes mellitus (T2DM) and to determine whether hyperglycemia moderates the association between regional cortical QSM values and cognitive performance.

**Methods:**

In this cross‐sectional study, 145 patients with T2DM and 182 healthy controls underwent 3 T MRI. Cortical gray matter magnetic susceptibility was quantified using quantitative susceptibility mapping (QSM). Cognitive function was assessed with a multidomain neuropsychological battery. Partial correlation, general linear models, and moderation analyses examined associations among cortical susceptibility, glycemic indices, and cognitive performance. Support vector regression (SVR) with nested cross‐validation evaluated the predictive value of limbic subregional QSM values for information processing speed.

**Results:**

Whole‐cortex analyses showed nominally higher QSM values in the bilateral limbic cortices of patients with T2DM, while post hoc analyses identified significantly higher QSM values in the left cingulate cortex and right parahippocampal gyrus after FDR correction. QSM values in selected limbic cortical regions at both the lobar and subregional levels were positively correlated with FPG and HbA1c. Covariate‐adjusted analyses further indicated associations between higher QSM values in frontal, parietal, and limbic regions and poorer episodic memory and executive function. Moderation analyses suggested that higher HbA1c levels strengthened the inverse associations between QSM values in selected limbic cortical regions and cognitive performance on the MMSE, MoCA, and CDT. Within the T2DM group, SVR models based on limbic subregional QSM values showed cross‐validated predictive performance for TMT‐A scores.

**Conclusions:**

Patients with T2DM showed a limbic‐predominant pattern of cortical magnetic susceptibility alterations, with higher QSM values in selected regions associated with poorer performance across multiple cognitive domains. HbA1c moderated the associations between regional QSM values and cognitive performance. These findings suggest that limbic cortical susceptibility alterations may be relevant to metabolic dysregulation and cognitive vulnerability in T2DM.

## Graphical abstract

Patients with type 2 diabetes show limbic‐predominant cortical magnetic susceptibility alterations associated with fasting glucose levels and poorer memory and executive function, suggesting that altered cerebral iron‐related susceptibility may represent a potential pathophysiological link between glycemic dysregulation and cognitive decline.

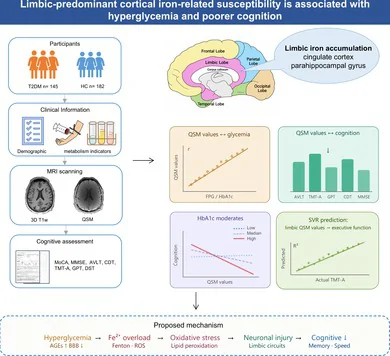

## 1 Introduction

Type 2 diabetes mellitus (T2DM) is a chronic metabolic disorder affecting hundreds of millions of individuals worldwide, with a health burden that extends well beyond classical complications such as cardiovascular and cerebrovascular disease [1]. In recent years, substantial epidemiological and clinical evidence has identified T2DM as an independent risk factor for mild cognitive impairment (MCI) and all‐cause dementia [2], particularly Alzheimer's disease (AD) [3], with estimates suggesting an approximately 1.5‐ to 2.5‐fold increase in dementia risk [4]. T2DM‐associated cognitive impairment is typically characterized in its early stages by deficits in executive function, reduced information processing speed, and impaired episodic memory [5, 6], each of which substantially compromises quality of life and imposes a considerable societal burden. Elucidating the neurobiological mechanisms underlying T2DM‐associated cognitive impairment is therefore critical for developing effective early intervention strategies.

Aberrant brain iron deposition has been closely implicated in several neurodegenerative diseases, including AD and Parkinson's disease (PD) [7, 8]. As one of the most iron‐rich organs in the body, the brain relies on iron as an essential cofactor for mitochondrial energy metabolism, myelin synthesis, and neurotransmitter production [9]. Cerebral iron accumulates progressively with aging, with the most prominent increases occurring in deep gray matter nuclei and cortical regions [10, 11]. Previous MRI‐based studies have reported increased iron content in deep gray matter nuclei, including the putamen, of patients with T2DM [12, 13]. However, the cerebral cortex serves as the central hub for higher‐order cognitive processing, and alterations within its microenvironment play a more direct role in cognitive decline. Recently, cortical iron has been increasingly investigated using both post‐mortem analysis [14] and in vivo imaging in healthy older adults [11], patients with cognitive impairment and those with AD [14, 15].

Quantitative susceptibility mapping (QSM) is an advanced MRI post‐processing technique that allows for the quantitative extraction of local tissue magnetic susceptibility values from phase information [16]. QSM demonstrates high specificity and sensitivity to paramagnetic substances, primarily non‐heme iron [17], and overcomes several confounds that limit the specificity of *R* ^2^* in brain regions, including contributions from myelin and calcium [16, 18]. The accuracy of QSM has been validated in several post‐mortem studies, which have established significant correlations between QSM contrast and histochemical measurements of iron in gray matter [19, 20].

The present study used QSM to systematically characterize regional patterns of cortical iron‐related magnetic susceptibility in patients with T2DM and to examine its associations with multidomain cognitive performance and glycemic measures. We hypothesized that [1] patients with T2DM would exhibit higher QSM values in selected cortical regions than healthy controls; [2] higher regional cortical QSM values would be associated with poorer cognitive performance, and [3] higher glycemic marker levels would moderate the associations between cortical QSM values and cognitive performance.

## 2 Materials and Methods

### 2.1 Participants and Study Design

This cross‐sectional observational study was approved by the local ethics committee. Right‐handed individuals aged 30–75 years were recruited for this study between January 2022 and December 2023. Written informed consent was obtained from all participants prior to enrollment. The diagnosis of T2DM was established in accordance with the American Diabetes Association (ADA) guidelines [21]; patients with a self‐reported physician‐diagnosed T2DM who were currently receiving hypoglycemic treatment were also included. Healthy controls (HC) were recruited from the hospital's physical examination center and were required to have a fasting plasma glucose (FPG) level of ≤ 6.1 mmol/L. Detailed exclusion criteria are provided in the Appendix E1 Briefly, individuals with neurological/psychiatric disorders, major systemic illnesses, or MRI contraindications were excluded.

### 2.2 Clinical and Demographic Data Collection

Demographic and clinical characteristics were collected for all participants, including age, sex, years of education, body mass index (BMI), disease duration, and blood pressure (BP). Microvascular risk factors and lifestyle habits, including history of hypertension, smoking status, and alcohol consumption, were also recorded. For biochemical analysis, all participants fasted for at least 12 h, and venous blood samples were collected at 08:00 the following morning. The metabolic biomarkers analyzed primarily encompassed: (i) glycemic indices: glycated hemoglobin (HbA1c), FPG, and fasting insulin (FINS); and (ii) lipid profiles: total cholesterol (TC), triglycerides (TG), high‐density lipoprotein cholesterol (HDL‐C), and low‐density lipoprotein cholesterol (LDL‐C).

### 2.3 Neuropsychological Assessment

All neuropsychological assessments were administered by rigorously trained professionals under standardized conditions. To ensure objectivity, the examiners were masked to the participants' medical history and group allocation. A comprehensive cognitive test battery was employed to cover distinct cognitive domains: (i) the Montreal Cognitive Assessment (MoCA) and Mini‐Mental State Examination (MMSE) screened for general cognitive impairment and assessed global cognitive status [22, 23]. (ii) The Auditory Verbal Learning Test (AVLT) evaluated immediate memory retention and delayed recall [24]. (iii) The Trail Making Test Part A (TMT‐A) [25], Grooved Pegboard Test (GPT) [26], and Clock Drawing Test (CDT) [27] assessed psychomotor speed and executive function. (iv) The Digit Span Test (DST) measured attention span and working memory [28].

### 2.4 MRI Acquisition

All participants were scanned using a 3.0‐Tesla Siemens MAGNETOM Prisma system (Siemens Healthcare, Erlangen, Germany) with a 64‐channel phased‐array head coil. Structural images were acquired using a three‐dimensional (3D) T1‐weighted MPRAGE sequence (voxel size: 1 × 1 × 1 mm^**3**^), and QSM data were collected using a 3D multi‐echo gradient‐recalled echo (mGRE) protocol (voxel size: 0.86 × 0.86 × 2 mm^3^). Detailed scanning parameters are provided in the (Appendix E2).

### 2.5 Image Processing

**FIGURE 1:**
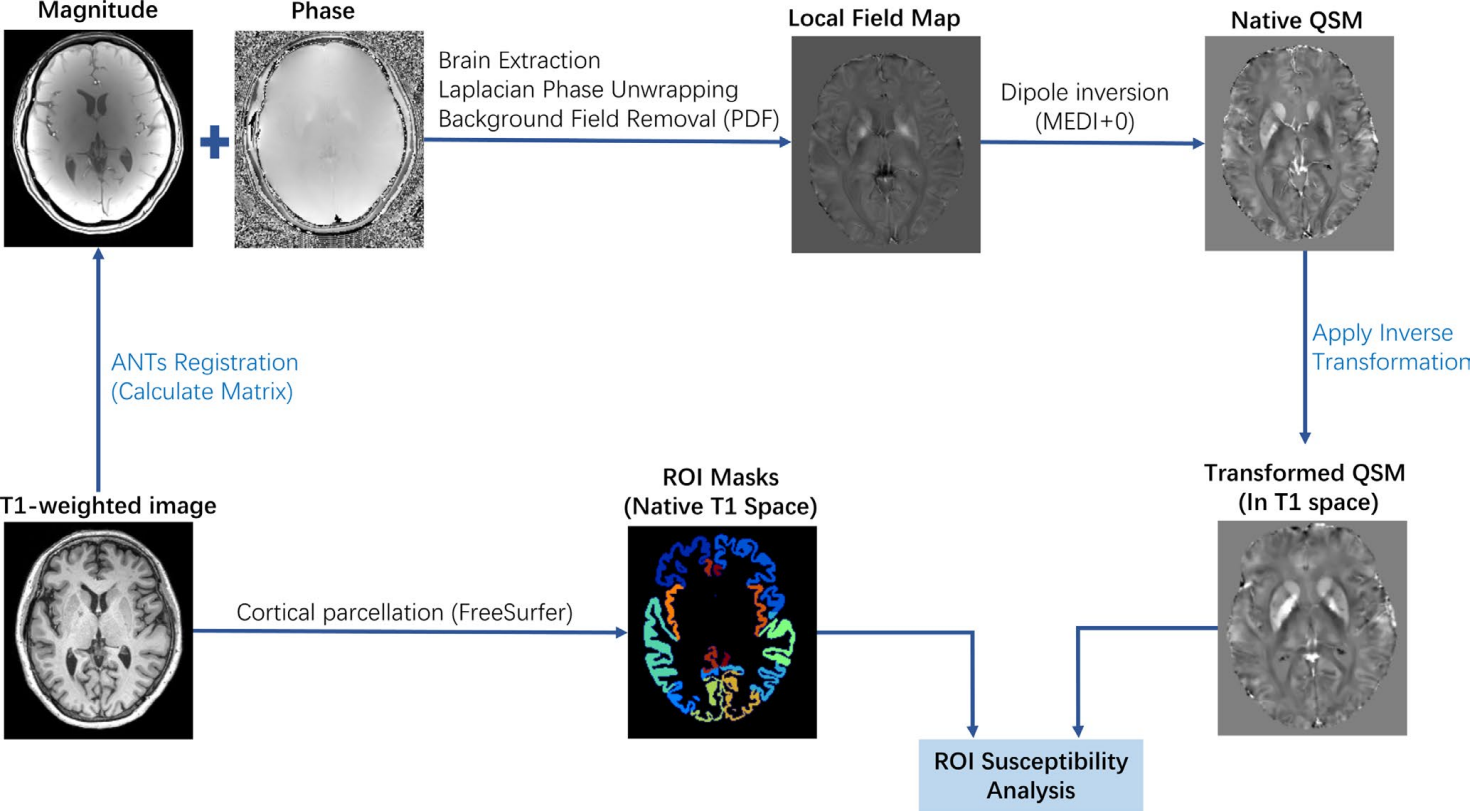
Pipeline overview of QSM preprocessing. Raw mGRE data were processed using the MEDI+0 pipeline to generate quantitative susceptibility maps. T1‐weighted images were segmented using FreeSurfer to define 12 cortical ROIs. The QSM images were then co‐registered and transformed into the T1 anatomical space for precise regional quantification. QSM, Quantitative susceptibility mapping; ROI, region of interest; mGRE, multi‐echo gradient‐recalled echo.

QSM data reconstruction was performed using the Morphology Enabled Dipole Inversion (MEDI+0) algorithm (available at https://pre.weill.cornell.edu/mri/pages/qsm.html) within the MATLAB R2021b environment. The processing pipeline comprised the following steps (Figure 1): (1) The total magnetic field map was derived by applying a nonlinear fit to the complex multi‐echo signals [29]; (2) spatial phase unwrapping guided by magnitude images to resolve frequency aliasing [30]; (3) separation of local tissue fields from background fields using the Projection onto Dipole Fields (PDF) method [31]; and (4) reconstruction of susceptibility maps using the MEDI inversion algorithm, with susceptibility values referenced to cerebrospinal fluid (CSF) as the zero baseline. This algorithm incorporates structural priors and a regularization term for ventricular CSF uniformity, effectively suppressing reconstruction artifacts and enhancing quantification accuracy [32].

For brain segmentation, the T1‐weighted MPRAGE images were co‐registered to the QSM space, and the FreeSurfer‐derived cortical parcellation labels were transformed into the QSM space using the resulting registration. Labels from the Desikan–Killiany atlas were combined to generate bilateral lobar ROI masks for the frontal, parietal, occipital, temporal, limbic, and insular cortices. For the post hoc analyses, six limbic subregional ROI masks were generated, comprising the bilateral entorhinal cortices, parahippocampal gyri, and cingulate cortices. Mean QSM values were extracted from each ROI as measures of regional tissue magnetic susceptibility. Detailed procedures for image registration, ROI definition, and quality control are provided in Appendix E3. The subject inclusion and image quality‐control flowchart and representative quality‐control examples are presented in Figures S1 and S2, respectively.

### 2.6 Statistical Analysis

Statistical analyses were conducted in R (version 4.3.1). Data are presented as mean ± SD or median (IQR) based on the Kolmogorov–Smirnov normality test. Group comparisons used chi‐square (*χ* ^2^) tests for categorical variables and Mann–Whitney *U* tests for continuous variables. Between‐group cortical QSM differences were assessed by ANCOVA, covarying for age, sex, and education. Given the nominal between‐group difference observed in the limbic cortex, exploratory subregional analyses were conducted across six bilateral limbic regions, comprising the left and right entorhinal cortices, parahippocampal gyri, and cingulate cortices. Partial Spearman correlation analyses, adjusted for age, sex, and years of education, were used to examine associations between regional QSM values and glycemic measures. Correction for multiple comparisons was performed using the Benjamini–Hochberg false discovery rate (FDR) procedure, with FDR‐adjusted *p* < 0.05 considered statistically significant. The statistical families for FDR correction were defined as follows: (i) between‐group comparisons across 12 lobar cortical ROIs; (ii) post hoc between‐group comparisons across 6 limbic subregions; and (iii) partial correlations between QSM values and glycemic indices, corrected separately within the lobar ROIs and the limbic subregions. Both uncorrected and FDR‐adjusted *p* values are reported for all analyses subject to multiple‐comparison correction.

Multiple linear regression models, adjusting for age, sex, education, BMI, and vascular risk factors (hypertension, smoking, and alcohol consumption), were used to evaluate associations between regional QSM values and cognitive performance, with uncorrected *p* < 0.05 used as the threshold for describing exploratory patterns of association.

To explore whether glycemic markers modulate the association between cortical QSM values and cognitive performance, moderation models were constructed in the full sample using HbA1c, FPG, and FINS as separate moderators. The regression model was specified as follows:

$$\begin{array}{c} \text{Cognitive score}=\beta_{0}+\beta_{1}\mathrm{QSM}\_c+\beta_{2}M\_c+\beta_{3}\;\left(\mathrm{QSM}\_c\times M\_c\right)\\ \;+\beta_{4}\text{Group}+\beta_{5}\;\left(\text{Group}\times \mathrm{QSM}\_c\right)+\beta_{6}\mathrm{Age}+\beta_{7}\mathrm{Sex}\\ \;+\beta_{8}\text{Education}+\varepsilon \end{array}$$

where *Y* denotes the cognitive score, QSM_c denotes the mean‐centered QSM value, M_c denotes the mean‐centered moderator (HbA1c, FPG, or FINS), Group denotes the diagnostic status (0 = HC, 1 = T2DM), and ε the residual error. The diagnostic group's main effect and the Group × QSM interaction term were included to control for baseline differences between T2DM and HC groups and for between‐group heterogeneity in the QSM‐cognition association. Variance inflation factors (VIFs) were computed for each model to evaluate potential multicollinearity among predictors. The maximum VIF across all terms in each model (VIF_*max*_) was reported, with values below 5 considered acceptable. The robustness of significant interaction effects was verified using 5000‐resample percentile bootstrap 95% confidence intervals. For interactions reaching significance (*p* < 0.05 with bootstrap 95% CI excluding zero), simple slope analyses were conducted at low (−1 SD), mean, and high (+1 SD) levels of the moderator to characterize the pattern of the interaction.

In the T2DM group, SVR (RBF kernel) predicted TMT‐A scores from limbic QSM values, with age, sex, and education as covariates. Nested five‐fold cross‐validation with inner‐fold grid search was used for hyperparameter tuning. Model performance was assessed by using RMSE, MAE, *R* ^2^, and Spearman's *r*, with 95% confidence intervals estimated via 1000‐iteration bootstrap resampling of the out‐of‐sample predictions. The statistical significance of *R* ^2^ was evaluated by permutation testing (*n* = 1000), and *p* values for both *R* ^2^ and Spearman's *r* were FDR‐corrected.

### 2.7 Sensitivity Analysis

To assess the robustness of our findings, a sensitivity analysis was performed. Although sex was included as a covariate in the primary analysis, a significant sex imbalance was present between groups. Propensity score matching (PSM) was therefore performed based on age, sex, education, and BMI. A 1:1 nearest‐neighbor matching algorithm with a caliper width of 0.25 was applied, yielding 113 matched participants per group. Standardized mean differences (SMDs) were calculated for all variables before and after matching to assess covariate balance. All primary analyses were then repeated in this PSM‐derived cohort.

### 2.8 Data and Resource Availability

Data are available by request from the corresponding author.

## 3 Results

### 3.1 Demographic and Clinical Characteristics

The study cohort comprised 327 participants, including 145 patients with T2DM (56.55% male, mean age: 52 years) and 182 HC (31.32% male, mean age: 52 years) (Table 1). Age and years of education were comparable between groups. However, a significant difference was observed in sex distribution (*p* < 0.001). The prevalence of hypertension was significantly higher in the T2DM group (35.86%) than in HC (6.04%, *p* < 0.001). As expected, patients with T2DM exhibited significantly higher levels of HbA1c, FPG, TG, along with significantly lower levels of HDL‐C. No significant intergroup differences were found in smoking history, alcohol consumption, BMI, or TC levels.

**TABLE 1:** Demographic, clinical and cognitive characteristics of study participants.

	HC (*n* = 182)	T2DM (*n* = 145)	Statistic	*p*
Demographics				
Age (years)	52.00 (43.00, 56.00)	52.00 (46.00, 60.00)	*Z* = −1.93	0.054
Sex (male/female)	57/125	85/60	*χ* ^2^ = 24.49	**< 0.001**
Education (years)	10.00 (8.00, 13.00)	11.00 (8.00, 14.00)	*Z* = −0.14	0.899
Diabetes duration (months)		48.00 (24.00, 120.00)		
Clinical data				
SBP (mmHg)	125.76 ± 18.78	128.43 ± 17.80	*t* = −1.29	0.199
DBP (mmHg)	80.00 (74.00, 89.00)	80.00 (73.00, 87.00)	*Z* = −0.57	0.568
BMI (kg/m^2^)	23.32 (21.77, 25.36)	23.66 (21.53, 26.66)	*Z* = −1.42	0.156
TC (mmol/L)	5.00 (4.50, 5.63)	4.90 (4.32, 5.59)	*Z* = −0.67	0.505
TG (mmol/L)	1.15 (0.84, 1.64)	1.56 (1.12, 2.19)	*Z* = −4.87	**< 0.001**
HDL‐C (mmol/L)	1.45 (1.23, 1.74)	1.15 (0.94, 1.34)	*Z* = −6.97	**< 0.001**
LDL‐C (mmol/L)	3.24 (2.77, 3.80)	3.18 (2.57, 3.82)	*Z* = −0.65	0.514
HbA1c (%)	5.70 (5.50, 5.90)	7.80 (6.60, 9.40)	*Z* = −13.15	**< 0.001**
FPG (mmol/L)	5.06 (4.83, 5.44)	7.29 (5.93, 9.29)	*Z* = −10.92	**< 0.001**
FINS (μIU/mL)	8.22 (5.65, 12.27)	7.17 (4.82, 12.00)	*Z* = −1.85	0.064
Vascular risk factor				
Smoking, *n* (%)	16 (8.79)	21 (15.22)	*χ* ^*2*^ = 3.17	0.075
Hypertension, *n* (%)	11 (6.04)	52 (35.86)	*χ* ^2^ = 46.13	**< 0.001**
Alcohol, *n* (%)	16 (8.79)	21 (15.22)	*χ* ^2^ = 3.17	0.075
Cognitive scores				
MoCA	26.00 (24.00, 28.00)	26.00 (23.00, 27.00)	*Z* = −1.60	0.109
MMSE	28.00 (27.00, 29.00)	28.00 (27.00, 29.00)	*Z* = −1.21	0.227
AVLT‐immediate	21.00 (19.00, 25.00)	21.00 (17.00, 24.00)	*Z* = −2.32	**0.020**
AVLT‐5 min	8.00 (7.00, 9.00)	8.00 (6.00, 9.00)	*Z* = −2.12	**0.034**
AVLT‐delay	8.00 (6.00, 10.00)	7.00 (5.00, 9.00)	*Z* = −2.61	**0.009**
AVLT‐recall	12.00 (11.00, 12.00)	12.00 (10.00, 12.00)	*Z* = −1.46	0.145
GPT‐R (s)	70.00 (61.00, 80.75)	71.50 (65.00, 90.00)	*Z* = −2.30	**0.022**
GPT‐L (s)	73.50 (65.00, 84.00)	80.00 (70.00, 95.00)	*Z* = −3.27	**0.001**
TMT‐A (s)	46.00 (32.00, 58.00)	51.00 (42.00, 66.00)	*Z* = −4.06	**< 0.001**
CDT	4.00 (3.00, 4.00)	4.00 (3.00, 4.00)	*Z* = −0.42	0.677
DST‐forward	7.00 (6.00, 8.00)	7.00 (6.00, 8.00)	*Z* = −0.89	0.374
DST‐backward	4.00 (3.00, 5.00)	4.00 (3.00, 5.00)	*Z* = −0.89	0.372

*Note:* Data are expressed as median (Q1, Q3) and mean ± SD. The categorical variables are expressed as number (percentage).

Abbreviations: AVLT, Auditory Verbal Learning Test; BMI, body mass index; CDT, Clock Drawing Test; DBP, diastolic blood pressure; DST, Digit Span Test; FINS, fasting insulin; FPG, fasting plasma glucose; GPT, Grooved Pegboard Test; HbA1c, hemoglobin A1c; LDL, low‐density lipoprotein; MMSE, Mini‐Mental State Examination; MoCA, Montreal Cognitive Assessment; SBP, systolic blood pressure; TC, total cholesterol; TG, triglycerides; TMT‐A, Trail Making Test‐A.

### 3.2 Cognitive Assessment Results

No significant between‐group differences were observed in MoCA or MMSE scores (both *p* > 0.05), indicating comparable global cognitive status. However, domain‐specific assessments revealed significant cognitive decline in the T2DM group. Specifically, on the AVLT, patients with T2DM exhibited significantly lower scores in immediate recall (*p* = 0.020), 5 min delayed recall (*p* = 0.034), and long‐delayed recall (*p* = 0.009) compared to healthy controls, indicating distinct impairments in episodic memory. On the GPT, which assesses fine motor skills and psychomotor speed, the T2DM group required significantly longer to complete the task with both hands (left: *p* = 0.001; right: *p* = 0.022). Similarly, T2DM patients demonstrated significantly prolonged TMT‐A completion times relative to HC (*p* < 0.001), indicating slowed information processing speed and executive function. Further details of the cognitive assessments are provided in Table 1.

### 3.3 Between‐Group Differences in Cortical Susceptibility and Limbic Subregional Analysis

After adjustment for age, sex, and years of education, comparisons across the 12 lobar cortical ROIs showed nominally higher susceptibility values in the left and right limbic cortices of the T2DM group (left: *t* = 2.203, *p* = 0.028, *p*FDR = 0.278; right: *t* = 2.000, *p* = 0.046, *p*FDR = 0.278). However, neither difference survived FDR correction across the 12 lobar ROIs, and no lobar‐level comparison remained statistically significant after correction (Table 2).

**TABLE 2:** QSM values for cortex ROIs in both T2DM and HC groups.

Region	T2DM	HC	Statistic	*p*	*P*FDR
Left frontal cortex	−6.65 (−12.08, −1.74)	−9.09 (−14.29, −4.41)	1.480	0.14	0.559
Right frontal cortex	−6.21 (−10.46, −1.31)	−8.43 (−13.19, −2.91)	1.314	0.19	0.565
Left parietal cortex	−0.65 (−6.76, 2.98)	−3.12 (−8.10, 1.81)	0.995	0.32	0.565
Right parietal cortex	−0.88 (−6.88, 3.07)	−2.45 (−6.88, 1.81)	0.322	0.748	0.816
Left temporal cortex	−8.73 (−13.13, −3.33)	−9.56 (−14.33, −3.38)	0.966	0.335	0.565
Right temporal cortex	−10.43 (−15.47, −5.98)	−12.00 (−18.08, −5.60)	0.644	0.52	0.693
Left occipital cortex	−1.77 (−7.31, 3.55)	−4.64 (−11.36, 2.31)	1.012	0.312	0.565
Right occipital cortex	−1.49 (−6.19, 4.35)	−3.65 (−10.54, 3.32)	0.886	0.376	0.565
Left insular cortex	−13.91 (−20.70, −8.79)	−15.76 (−22.01, −8.95)	0.178	0.859	0.859
Right insular cortex	−11.94 (−17.88, −4.71)	−13.30 (−19.42, −6.94)	0.334	0.738	0.816
Left limbic cortex	−2.17 (−7.73, 4.12)	−6.17 (−14.08, 0.40)	2.203	**0.028**	0.278
Right limbic cortex	−2.60 (−7.90, 2.90)	−6.14 (−13.73, 0.60)	2.000	**0.046**	0.278

*Note:* Significant results (*p* < 0.05) are reported in bold.

**FIGURE 2:**
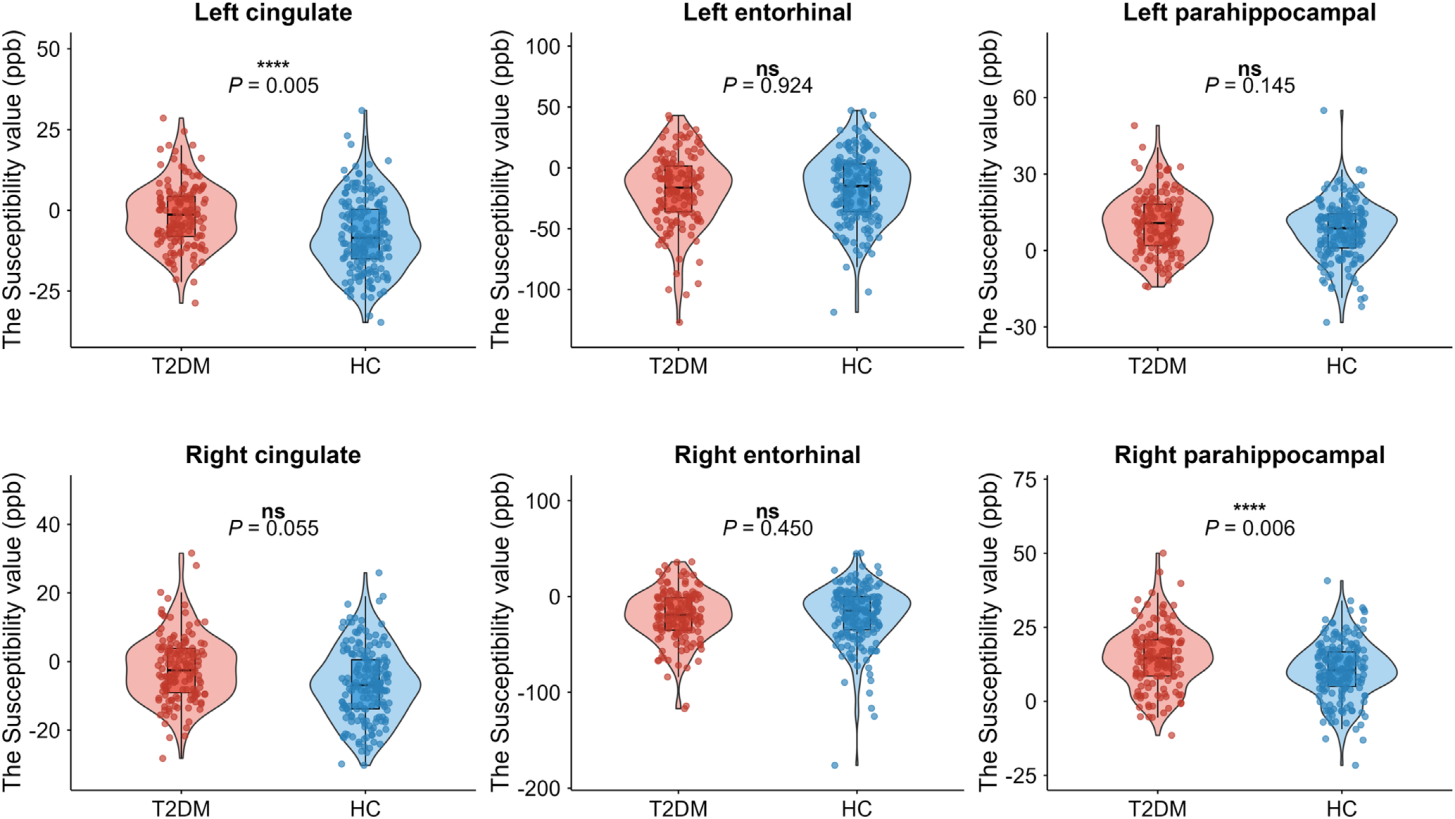
Violin plots illustrating differences in QSM values across limbic subregions between the T2DM and HC groups. *p < 0.05, **p < 0.01, ***p < 0.001, ****pFDR < 0.05.

Post hoc analyses were conducted across six limbic subregions to examine whether the observed nominal differences were localized to specific subregions. After FDR correction across the six limbic subregions, the T2DM group showed significantly higher susceptibility in the left cingulate cortex (*t* = 2.849, *p* = 0.005, *p*FDR = 0.019) and right parahippocampal gyrus (*t* = 2.747, *p* = 0.006, *p*FDR = 0.019; Figure 2 and Table S1). No significant group differences were observed in the bilateral entorhinal cortices after FDR correction (*p*FDR > 0.05).

### 3.4 Correlations Between Cortical Susceptibility and Glycemic Metabolic Indices

**FIGURE 3:**
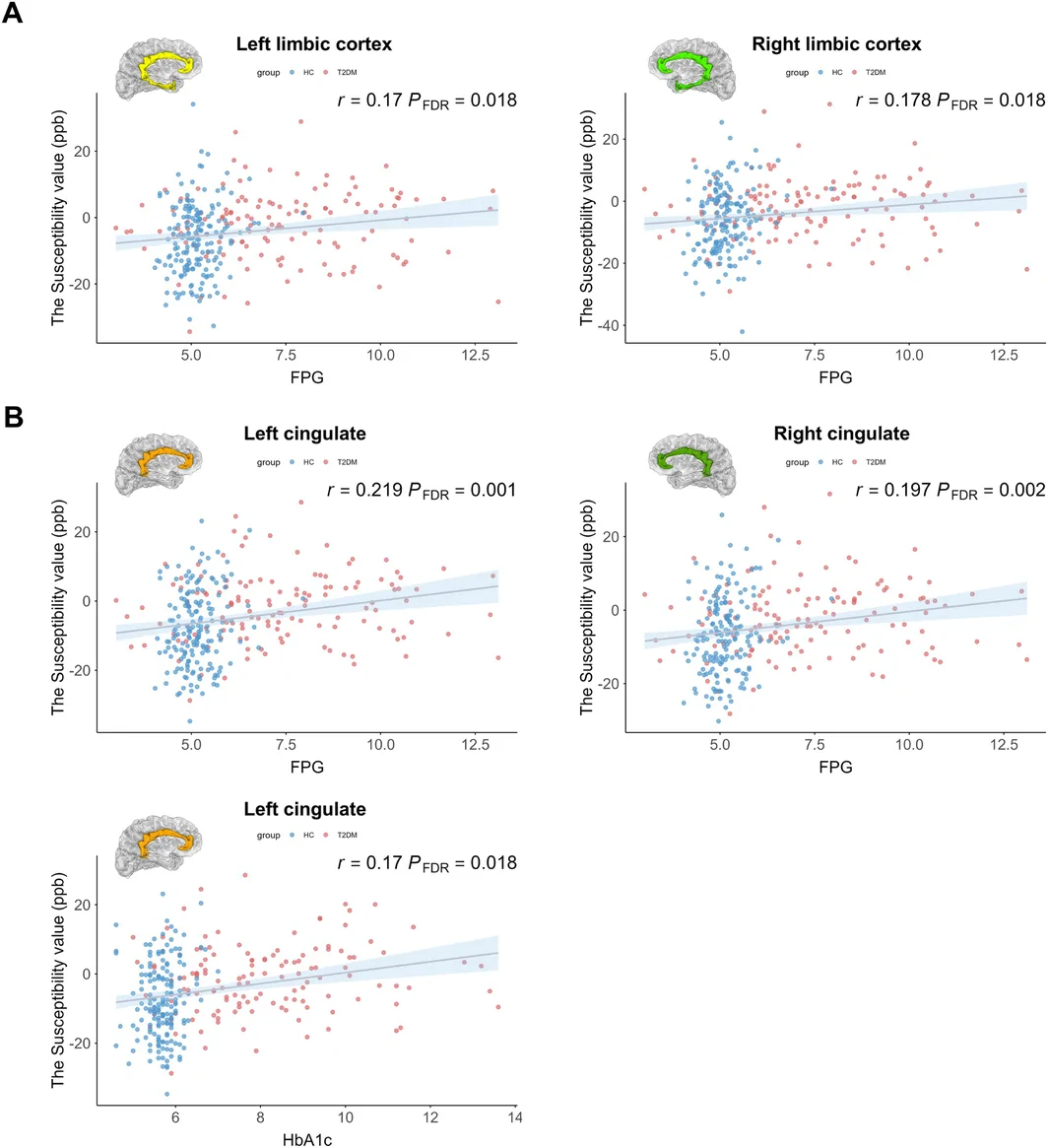
Correlations between cortical QSM values and glucose metabolism indicators. Scatter plots showing the linear relationships between QSM values and glucose metabolism indicators: (A) correlation between QSM values in limbic cortical regions and FPG; (B) correlation between QSM values in cingulate cortex and FPG and HbA1c. Detailed correlation statistics are shown in Table S2.

Across the entire cohort, Spearman partial correlation analyses revealed significant positive associations between limbic cortical QSM values and glycemic indices (Figure 3A,B, Tables S2 and S3). FPG was positively correlated with QSM values in the bilateral limbic cortices (left: *r* = 0.170, *p* = 0.003, *p*FDR = 0.018; right: *r* = 0.178, *p* = 0.002, *p*FDR = 0.018); sub‐regional analysis revealed that this association was most pronounced in the cingulate cortex (left: *r* = 0.219, *p* < 0.001, *p*FDR = 0.001; right: *r* = 0.197, *p* = 0.001, *p*FDR = 0.002). Similarly, HbA1c levels were positively correlated with susceptibility in the left cingulate cortex (*r* = 0.170, *p* = 0.003, *p*FDR = 0.018).

### 3.5 Independent Associations Between Cortical Susceptibility and Cognitive Domains

**FIGURE 4:**
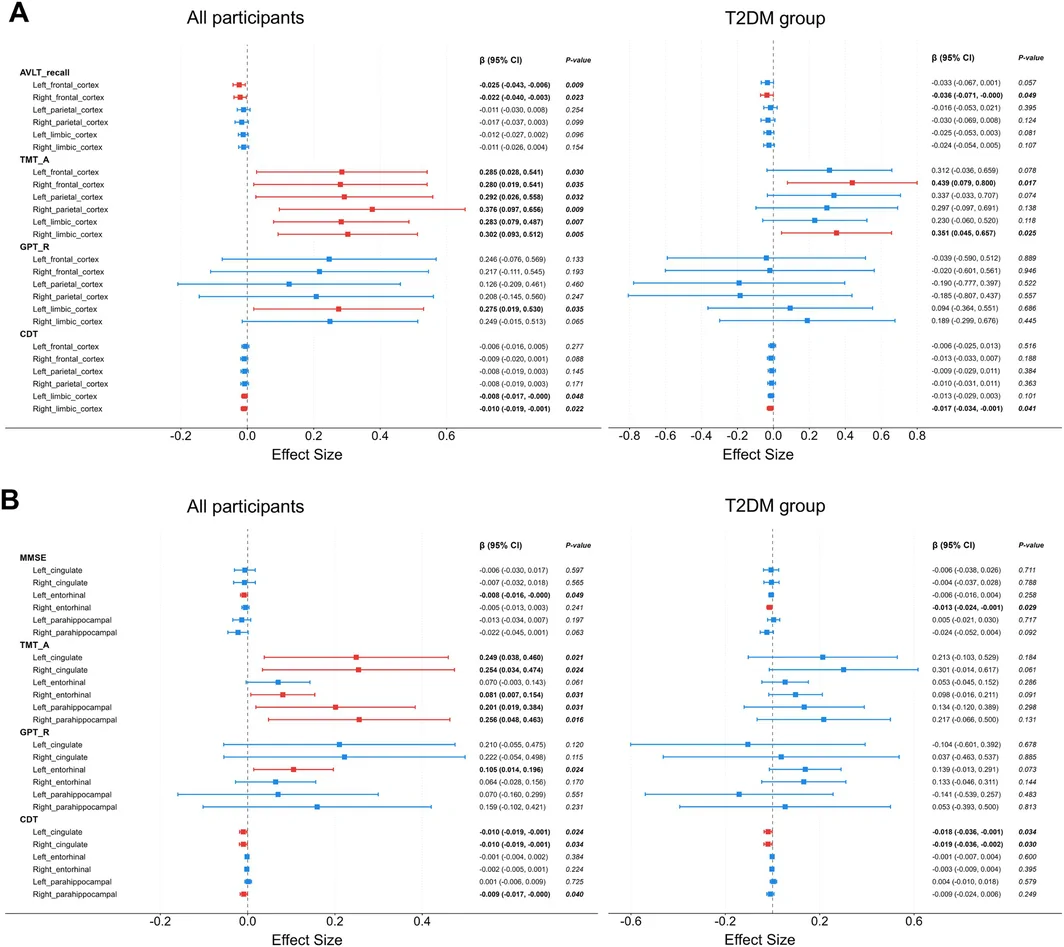
Results of multiple linear regression analysis. (A) Relationships between QSM values in cortical regions and multi‐domain cognitive scores. (B) Relationships between QSM values in limbic system subregions and multi‐domain cognitive scores. In the figure, red represents statistically significant findings (*p* < 0.05), and blue represents nonsignificant findings (*p* ≥ 0.05). Values in parentheses represent 95% confidence intervals (CI). Results for the bilateral temporal, occipital, and insular cortices are omitted due to the lack of significant associations between cortical iron and cognition in these regions. For the complete list of model coefficients, please refer to Tables S4–S7.

After adjusting for age, sex, education, BMI, and vascular risk factors (hypertension, smoking, and alcohol consumption), multivariate linear regression analyses revealed associations between cortical susceptibility and performance across multiple cognitive domains (*p* < 0.05; Figure 4A,B, Tables S4–S7).

#### 3.5.1 Memory and Cognitive Function

Higher QSM values in the bilateral frontal cortices were associated with lower AVLT recall scores (left: *β* = 10.025, *p* = 0.009; right: *β* = −0.022, *p* = 0.023). At the limbic subregional level, higher QSM values in the left entorhinal cortex were associated with lower MMSE scores (*β* = −0.008, *p* = 0.049). Within the T2DM subgroup, higher QSM values in the right frontal cortex were associated with lower AVLT recall scores (*β* = −0.036, *p* = 0.049), while higher right entorhinal QSM values were associated with lower MMSE scores (*β* = −0.013, *p* = 0.029).

#### 3.5.2 Information Processing Velocity and Executive Function

Higher QSM values across fronto‐parietal‐limbic regions were associated with longer TMT‐A completion times. Associations were observed in the right limbic cortex (*β* = 0.302, *p* = 0.005), right parietal cortex (*β* = 0.376, *p* = 0.009), bilateral frontal cortices, left parietal cortex, and left limbic cortex (all *p* < 0.05). At the limbic subregional level, higher QSM values in the bilateral cingulate cortices (left: *β* = 0.249, *p* = 0.021; right: *β* = 0.254, *p* = 0.024), right entorhinal cortex (*β* = 0.081, *p* = 0.031), and bilateral parahippocampal gyri (left: *β* = 0.201, *p* = 0.031; right: *β* = 0.256, *p* = 0.016) were associated with longer TMT‐A completion times. Within the T2DM subgroup, similar associations were observed in the right frontal cortex (*β* = 0.439, *p* = 0.017) and left limbic cortex (*β* = 0.351, *p* = 0.025).

#### 3.5.3 Fine Motor Skills (GPT‐R)

Higher QSM values in the left limbic cortex were associated with longer GPT right‐hand completion times (*β* = 0.275, *p* = 0.035). At the subregional level, a similar association was observed in the left entorhinal cortex (*β* = 0.105, *p* = 0.024).

#### 3.5.4 Executive and Visuospatial Function

Higher QSM values in the bilateral limbic cortices were associated with lower CDT scores (left: *β* = −0.008, *p* = 0.048; right: *β* = −0.010, *p* = 0.022). Subregional analysis indicated that these associations were most pronounced in the bilateral cingulate cortices (left: *β* = −0.010, *p* = 0.024; right: *β* = −0.010, *p* = 0.034), with similar associations observed in the right parahippocampal gyrus (*β* = −0.009, *p* = 0.040). Within the T2DM subgroup, higher QSM values in the bilateral cingulate cortices remained associated with lower CDT scores (left: *β* = −0.018, *p* = 0.034; right: *β* = −0.019, *p* = 0.030).

### 3.6 Moderating Effect of HbA1c on the Association Between Regional QSM Values and Cognitive Performance

After controlling for diagnostic group and Group × QSM interaction effects, moderation analyses revealed that HbA1c moderated the association between cortical QSM values and cognitive performance across multiple domains (all VIF_*max*_ < 5; Tables S8, S9).

**FIGURE 5:**
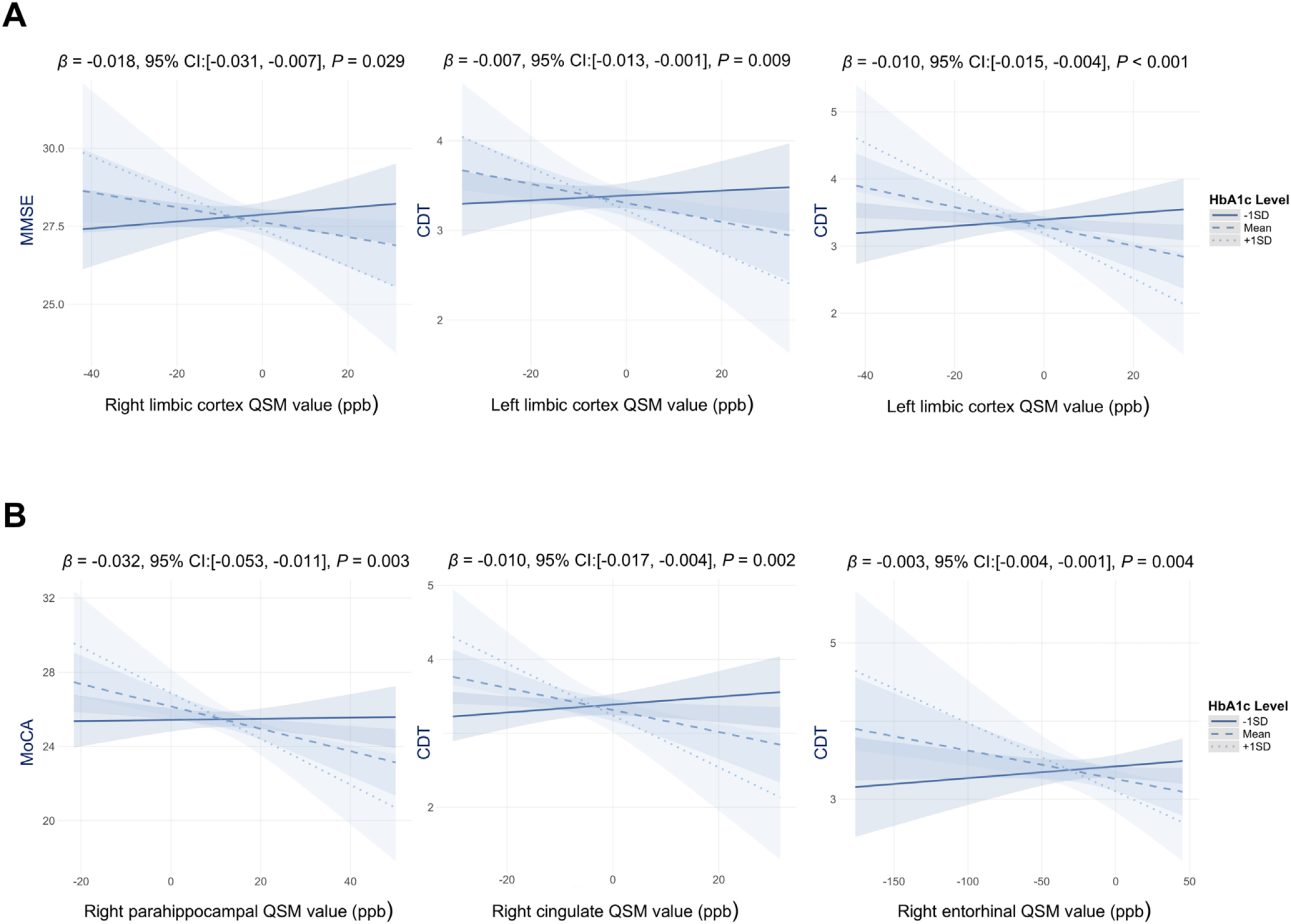
Simple slope analysis of the moderating effect of HbA1c on the association between limbic cortical QSM values and cognitive performance. The association between QSM values and cognitive scores is plotted at low (−1 SD), mean, and high (+1 SD) levels of HbA1c. (A) Lobar‐level limbic cortex. (B) Limbic subregions (cingulate, parahippocampal, and entorhinal cortices).

At the lobar level (Figure 5A), HbA1c moderated the association between right limbic cortex QSM values and MMSE scores (*β* = −0.018, 95% CI [−0.031, −0.007], *p* = 0.029), as well as the associations between bilateral limbic cortex QSM values and CDT scores (right: *β* = −0.010, 95% CI [−0.015, −0.004], *p* < 0.001; left: *β* = −0.007, 95% CI [−0.013, −0.001], *p* = 0.009). At the limbic subregional level (Figure 5B), HbA1c moderated the associations between right parahippocampal gyrus QSM values and MoCA scores (*β* = −0.032, 95% CI [−0.053, −0.011], *p* = 0.003), and between right cingulate cortex (*β* = −0.010, 95% CI [−0.017, −0.004], *p* = 0.002) and right entorhinal cortex (*β* = −0.003, 95% CI [−0.004, −0.001], *p* = 0.004) QSM values and CDT scores. All interaction effects were verified by 5000‐resample bootstrap 95% confidence intervals excluding zero.

Simple slope analyses revealed a consistent pattern across all models (Table 3): at low HbA1c levels (−1 SD), no association was observed between regional QSM values and cognitive performance (all *p* > 0.40), whereas at high HbA1c levels (+1 SD), higher QSM values were associated with poorer cognitive scores (all *p* < 0.05). For example, the association between right limbic cortex QSM and CDT scores was absent at low HbA1c (slope = 0.005, *p* = 0.431) but pronounced at high HbA1c (slope = −0.034, *p* = 0.001). Similarly, the association between right parahippocampal QSM and MoCA scores was non‐significant at low HbA1c (slope = 0.003, *p* = 0.884) but emerged at high HbA1c (slope = −0.124, *p* = 0.002). These findings suggest that chronic hyperglycemia, as indexed by elevated HbA1c, may amplify the adverse effect of cortical iron accumulation on cognitive function, particularly in the limbic system.

**TABLE 3:** Associations QSM values and cognitive scores at different levels of HbA1c.

			95% CI	*p*	
	Moderator Level (HbA1c)	Slope	Lower	Upper	
MMSE					
Right limbic cortex	Low (−1 SD)	0.011	−0.023	0.045	0.520
	Mean	−0.024	−0.059	0.011	0.184
	High (+1 SD)	−0.059	−0.116	−0.001	**0.045**
CDT					
Left limbic cortex	Low (−1 SD)	0.003	−0.009	0.015	0.653
	Mean	−0.011	−0.023	0.002	0.092
	High (+1 SD)	−0.024	−0.043	−0.005	**0.014**
Right limbic cortex	Low (−1 SD)	0.005	−0.007	0.017	0.431
	Mean	−0.014	−0.027	−0.002	**0.024**
	High (+1 SD)	−0.034	−0.054	−0.013	**0.001**
MoCA					
Right parahippocampal	Low (−1 SD)	0.003	−0.039	0.045	0.884
	Mean	−0.060	−0.106	−0.015	**0.010**
	High (+1 SD)	−0.124	−0.201	−0.047	**0.002**
CDT					
Right cingulate	Low (−1 SD)	0.005	−0.007	0.018	0.406
	Mean	−0.015	−0.028	−0.001	**0.031**
	High (+1 SD)	−0.035	−0.058	−0.012	**0.003**
Right entorhinal	Low (−1 SD)	0.002	−0.003	0.006	0.462
	Mean	−0.004	−0.008	0.001	0.087
	High (+1 SD)	−0.009	−0.015	−0.002	**0.009**

*Note:* Significant results (*p* < 0.05) are reported in bold.

### 3.7 Prediction of Information‐Processing Speed From Limbic Subregional QSM Values

**FIGURE 6:**
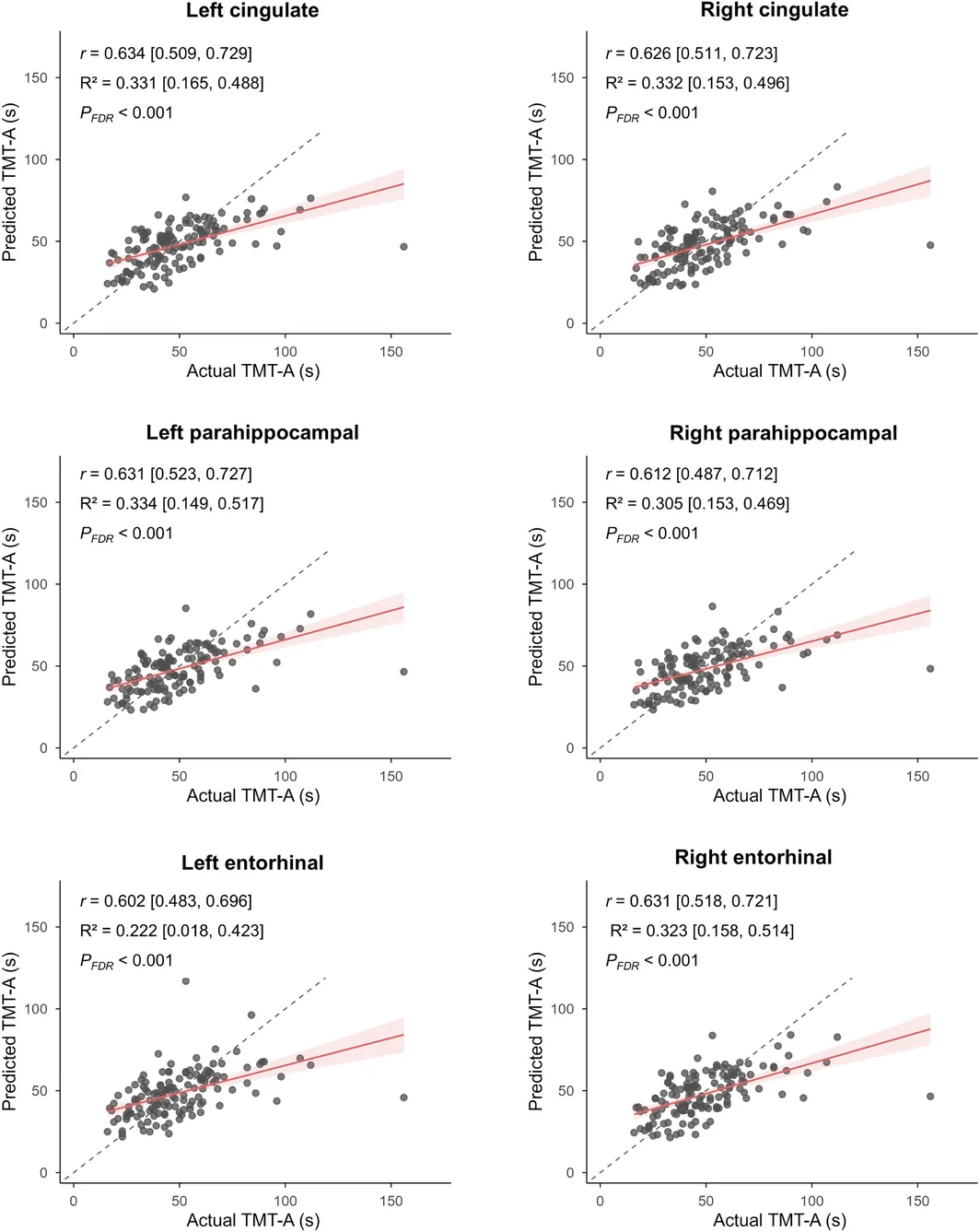
Prediction of TMT‐A scores in patients with T2DM. Scatter plots show the linear relationship between the predicted and measured clinical TMT‐A scores, with the Spearman correlation coefficient (*r*), coefficient of determination (*R* ^2^), and their corresponding 95% confidence intervals reported to assess predictive performance.

SVR modeling demonstrated that QSM values in limbic subregions significantly predicted TMT‐A performance in the T2DM group (Figure 6). The strongest predictive associations were observed for the bilateral cingulate cortices (left: *r* = 0.634, *R* ^2^ = 0.331; right: *r* = 0.626, *R* ^2^ = 0.332), followed by the bilateral parahippocampal gyri (left: *r* = 0.631, *R* ^2^ = 0.334; right: *r* = 0.612, *R* ^2^ = 0.305) and bilateral entorhinal cortices (left: *r* = 0.602, *R* ^2^ = 0.222; right: *r* = 0.631, *R* ^2^ = 0.323), with all models surviving FDR correction (all *p*FDR < 0.001). Bootstrap 95% confidence intervals for all performance metrics are reported in Table S10. These results indicate that limbic subregional susceptibility values explain approximately 30% of the variance in TMT‐A performance in T2DM.

### 3.8 Sensitivity Analysis

Following PSM, a matched cohort of 226 participants was constructed, with 113 participants in each group. Baseline characteristics and standardized mean differences (SMDs) before and after matching are presented in Table S11. Analyses conducted in the PSM cohort largely supported the primary findings, with detailed results provided in Appendix E4, Figures S3–S7, and Tables S12–S22. Matching improved overall covariate balance, with satisfactory balance achieved for age, sex, education, and BMI, although residual imbalance remained for several metabolic and cardiovascular variables. The handling of these residual imbalances, including additional covariate‐adjusted analyses, is detailed in Appendix E5, with the corresponding results presented in Figure S8 and Table S23.

## 4 Discussion

We identified a limbic‐predominant pattern of higher cortical magnetic susceptibility in T2DM, particularly involving the cingulate cortex and parahippocampal gyrus. Regional QSM values were positively associated with glycemic indices and with poorer performance across multiple cognitive domains, while HbA1c moderated several QSM–cognition associations. In addition, SVR modeling demonstrated that limbic subregional QSM values predicted information‐processing speed. Together, these findings suggest that cortical susceptibility alterations are associated with metabolic dysregulation and cognitive performance in T2DM.

### 4.1 Regional Cortical QSM Differences and Glycemic Associations

At the lobar level, nominally higher QSM values were observed in the bilateral limbic cortices of the T2DM group, although these differences did not survive FDR correction across the 12 lobar ROIs. Post hoc subregional analyses identified significantly higher QSM values in the left cingulate cortex and right parahippocampal gyrus after FDR correction, consistent with a previous QSM study reporting higher magnetic susceptibility in the right parahippocampal gyrus in T2DM [33]. The parahippocampal gyrus is a pivotal limbic memory hub; its early impairment in AD and MCI is closely linked to episodic memory deficits [34]. Moreover, the cingulate gyrus serves a critical role at the limbic‐neocortical interface, integrating emotional regulation with executive control and attentional processing [35, 36]. Higher magnetic susceptibility in this region may reflect iron‐related tissue alterations that could potentially affect the functional integrity of the default mode network (DMN) or the salience network, potentially contributing to the attentional deficits and psychomotor slowing frequently observed in T2DM.

Correlation analyses demonstrated associations between glycemic indices and regional cortical magnetic susceptibility. FPG levels exhibited significant positive correlations with susceptibility in the bilateral limbic system. This finding is consistent with previous neuroimaging literature. Previous QSM‐based studies have reported associations between poor glycemic control and higher magnetic susceptibility in brain regions such as the hippocampus and basal ganglia [12, 13]. Subregional analysis pinpointed the cingulate cortex as the main contributor to these limbic correlations. As a critical hub integrating metabolic signals [37], the cingulate cortex (especially the anterior cingulate cortex) is known to be sensitive to glucose and insulin variations [38, 39]. Beyond its central role in episodic, contextual, and emotional memory [36], the limbic system is highly susceptible to metabolic disturbances and oxidative stress [40, 41]. While iron serves as an essential cofactor for mitochondrial oxidative phosphorylation, myelin synthesis, and neurotransmitter synthesis [9, 42], its excess can catalyze the production of reactive oxygen species (ROS) via the Fenton reaction, leading to oxidative stress, lipid peroxidation, and cell death [43].

Several mechanisms may link hyperglycemia to iron‐related alterations in tissue susceptibility. The accumulation of advanced glycation end products (AGEs) induced by hyperglycemia can upregulate the receptor for AGEs (RAGE), thereby promoting cellular iron uptake and inhibiting iron efflux [44]. In addition, the systemic inflammatory state and blood–brain barrier (BBB) dysfunction caused by chronic hyperglycemia may compromise barrier integrity, leading to increased permeability and the subsequent infiltration of circulating transferrin‐bound or non‐transferrin‐bound iron into the brain parenchyma [45, 46]. Finally, insulin resistance may impair the regulation of iron storage and transport proteins [47], further exacerbating iron dyshomeostasis. Collectively, these alterations have been associated with expanded pools of labile iron and elevated iron‐mediated oxidative stress, particularly in metabolically vulnerable regions such as the basal ganglia, cortex, and hippocampus. The synergistic interplay of oxidative stress, inflammation, and iron metabolic disruption may precipitate neurodegenerative processes [48]. This convergence of pathways may thus represent a structural substrate for the memory deficits observed in T2DM under conditions of chronic hyperglycemia and insulin resistance.

### 4.2 Potential Mechanisms Underlying the Associations of Cortical QSM Values With Memory and Executive Function

Elevated magnetic susceptibility in the frontal lobe was associated with poorer AVLT recall performance, which may be related to alterations in executive control networks involved in memory retrieval and organization. This interpretation is consistent with findings in amyloid‐β‐negative populations, in which greater regional iron burden, including in the frontal cortex, was associated with poorer global cognition, episodic memory, and executive function [49]. Previous research has established that the frontal cortex exerts critical top‐down modulation during the encoding and strategic retrieval of episodic memory [50].

The broader pattern of higher QSM values across fronto‐parietal‐limbic circuits was associated with psychomotor slowing and executive dysfunction. This aligns with a prior voxel‐based QSM study linking susceptibility in the right frontal inferior triangular, middle frontal, and inferior frontal gyri to TMT‐A performance in T2DM [51]. Sub‐regional analysis further revealed that QSM values in the cingulate cortex, entorhinal cortex, and parahippocampal gyrus showed consistent associations with executive deficits. These regions correspond to the sites of earliest tau protein aggregation and neuronal loss in the pathological progression of AD [52, 53]. This spatial overlap suggests that the regional cortical susceptibility alterations observed in T2DM may indicate overlapping patterns of vulnerability to neurodegenerative processes, providing a possible neurobiological context for the established association between T2DM and increased dementia risk.

Abnormal iron accumulation has been proposed to affect cortical microstructural integrity and neural conduction through mechanisms involving oxidative stress and oligodendrocyte injury [54], and increased cortical iron burden has also been reported in association with cognitive impairment in AD and MCI cohorts [55]. Whereas previous studies focusing on the basal ganglia have demonstrated associations between iron deposition and psychomotor slowing [12], the present QSM findings extend these observations to cortical networks and suggest that cortical susceptibility alterations may also be relevant to cognitive slowing in T2DM.

### 4.3 Moderating Role of HbA1c in Cortical QSM and Cognition Associations

Moderation analyses revealed that, after controlling for diagnostic group effects, HbA1c—an index of average glycemic exposure over the preceding 2–3 months—moderated the association between limbic cortical susceptibility and performance across multiple cognitive domains. At higher HbA1c levels, the associations between QSM values in the cingulate cortex, entorhinal cortex, and parahippocampal gyrus and poorer visuospatial/executive function (CDT) and memory performance (MoCA, MMSE) were more pronounced, whereas no such associations were observed at lower HbA1c levels. These findings suggest that metabolic dysregulation and brain iron accumulation may jointly contribute to cognitive vulnerability. Beyond the AGE‐related pathway noted above, hyperglycemia independently promotes oxidative and endoplasmic reticulum stress through polyol pathway activation [56], hexosamine pathway enhancement [57], and protein kinase C activation [58]. When these pathways coexist with iron‐related oxidative stress and mitochondrial dysfunction [43], they may synergistically collapse antioxidant defenses and accelerate neuronal damage. This pattern is consistent with AD models in which insulin resistance accelerates iron‐related plaque deposition and cognitive decline [45]. Notably, moderation effects were concentrated in limbic subregions, aligning with the limbic‐predominant pattern of susceptibility alterations observed in the present study and further supporting the central role of these regions in T2DM‐related cognitive impairment. Given the exploratory nature of these analyses and the cross‐sectional design, these findings should be interpreted with caution and require validation in longitudinal studies.

### 4.4 Cross‐Validated Predictive Performance of Limbic Subregional QSM Values for Information‐Processing Speed

SVR modeling results provide convergent evidence for the associations identified in the regression analyses, demonstrating that limbic cortical QSM values significantly predicted TMT‐A performance in the T2DM group. Among the limbic subregions examined, the entorhinal cortex emerged as the strongest predictor, consistent with evidence that higher magnetic susceptibility in the entorhinal cortex is associated with an increased risk of developing mild cognitive impairment [49]. The use of SVR with nested cross‐validation strengthens confidence in these brain‐behavior associations by accommodating nonlinear relationships and reducing overfitting.

### 4.5 Limitations

Several limitations should be acknowledged. First, the cross‐sectional design precludes determination of the temporal or causal relationships among regional magnetic susceptibility, hyperglycemia, and cognitive performance. Second, QSM quantifies tissue magnetic susceptibility and is sensitive to paramagnetic iron but does not directly measure tissue iron concentration. QSM values may also be influenced by other susceptibility sources, including myelin and calcium. Third, merging cortical gyri from the Desikan‐Killiany atlas into lobar regions may have obscured spatially localized alterations. Fourth, the absence of peripheral iron‐related biomarkers, such as ferritin, transferrin saturation, and hepcidin, limited our ability to examine the relationship between peripheral and central iron metabolism. Fifth, the limbic subregional analyses were conducted post hoc based on lobar‐level findings and, despite surviving FDR correction, require independent validation. Future longitudinal studies integrating multimodal imaging, peripheral iron‐related biomarkers, and mechanistic assessments of iron transport are needed to clarify the temporal and biological relationships among glycemic dysregulation, regional susceptibility, and cognition. Independent validation and interventional studies will also be necessary to determine the reproducibility and potential clinical relevance of these findings.

## 5 Conclusion

Regional cortical magnetic susceptibility alterations in T2DM showed a limbic‐predominant spatial pattern and were associated with multidomain cognitive performance. Moderation analyses suggested that chronic hyperglycemia, as indexed by HbA1c, may strengthen the associations between limbic QSM values and cognitive impairment. Limbic QSM values further demonstrated predictive utility for information‐processing speed. Collectively, these findings suggest that QSM captures limbic susceptibility alterations relevant to metabolic status and cognitive performance in T2DM. Whether these susceptibility alterations specifically reflect changes in tissue iron requires confirmation using complementary imaging, biochemical, or histological measures.

## Author Contributions

Kun Wang contributed to study design, data analysis, methodology, software implementation, and manuscript drafting. Dan Hu contributed to study design and manuscript review. Jiahe Wang, Ziyu Diao, and Yuna Chen contributed to data curation and supervision. Xuan Huang and Die Shen contributed to data curation and formal analysis. Jie An and Shijun Qiu contributed to funding acquisition and manuscript revision. Gang Li contributed to study conception and design, data analysis, software implementation, supervision, and manuscript revision. Guarantors: Gang Li and Shijun Qiu are the guarantors of this work and take responsibility for the integrity of the data and the accuracy of the data analysis.

## Funding

This work was funded by the National Natural Science Foundation of China (NSFC), including Key Program grants [Nos. T2341014 and 82330058 (to Shijun Qiu)] and a General Program grant [No. 82471942 (to Jie An)], and the State Key Laboratory of Traditional Chinese Medicine Syndrome Projects [No. SKLKY2026C0001 (to Shijun Qiu)].

## Ethics Statement

This research was carried out following the principles set forth in the Declaration of Helsinki and was approved by the Institutional Review Board at the First Affiliated Hospital of Guangzhou University of Chinese Medicine (approved number: K‐2025–021).

## Consent

Written informed consent was acquired from all participants after a thorough explanation of the experimental procedures. Participants were clearly informed of their right to withdraw from the study at any point without any repercussions.

## Conflicts of Interest

The authors declare no conflicts of interest.

## Supporting information


**Appendix E1** Detailed exclusion criteria.
Appendix E2. MRI data acquisition.
Appendix E3. Anatomical parcellation and registration.
Appendix E4. Sensitivity analysis results.
Appendix E5. Additional adjustment for residual imbalance after PSM.
**Figure S1:** Subject inclusion and image quality control flowchart. Overview of the screening and exclusion process for image quality control, FreeSurfer cortical segmentation, and QSM‐to‐T1w registration. Numbers on the right at each stage indicate the number of subjects excluded. The final analytic sample comprised 145 patients with T2DM and 182 healthy controls.
**Figure S2:** Representative quality control examples. (A) FreeSurfer cortical segmentation quality, with pial surfaces (red) and white matter surfaces (yellow) overlaid on the T1‐weighted images in the axial plane. (B) QSM‐to‐T1w registration accuracy, with the co‐registered QSM maps overlaid on the T1‐weighted images. (C) Cortical parcellation labels based on the Desikan–Killiany atlas overlaid on the T1‐weighted images.
**Figure S3:** Violin plots illustrating differences in QSM values across limbic subregions between the T2DM and HC groups in the PSM cohort.
**Figure S4:** Correlations between cortical QSM values and glucose metabolism indicators in the PSM cohort. (A) Correlations between limbic cortical gray matter QSM values and glucose metabolism indicators. (B) Correlations between cingulate gray matter QSM values and glucose metabolism indicators.
**Figure S5:** Results of multiple linear regression analysis in PSM cohort. Relationships between QSM values in cortical regions and multi‐domain cognitive scores. Values in parentheses represent 95% confidence intervals (CI). Statistical significance is defined as p < 0.05. For the complete list of model coefficients, please refer to Tables S16–19.
**Figure S6:** Simple slope analysis plots in the PSM cohort. The association between QSM values and cognitive scores is plotted at low (−1 SD), mean, and high (+1 SD) levels of HbA1c. Detailed results of the moderation analyses and simple slope analyses are provided in Tables 20–21.
**Figure S7:** Prediction of TMT‐A scores in patients with T2DM in PSM cohort. Scatter plots show the linear relationship between the predicted and measured clinical TMT‐A scores, with the Spearman correlation coefficient (r), coefficient of determination (R2), and their corresponding 95% confidence intervals reported to assess predictive performance.
**Figure S8:** Between‐group comparisons of limbic subregional QSM values in the PSM‐matched cohort after additional adjustment for DBP, HDL‐C, and hypertension. Violin plots with overlaid box plots and individual data points. *p* values are from ANCOVA adjusting for age, sex, education, DBP, HDL‐C, and hypertension.
**Table S1:** QSM values for limbic cortex ROIs in both T2DM and HC groups.
**Table S2:** Relationship between cortical QSM values and glycemic metabolic profiles in all participants.
**Table S3:** Partial correlations between QSM values and glycemic metabolic indices in all participants.
**Table S4:** Relationships between QSM values in cortical regions and multi‐domain cognitive scores in all participants: multiple linear regression analysis.
**Table S6:** Relationships between QSM values in limbic system subregions and multi‐domain cognitive scores in all participants: multiple linear regression analysis.
**Table S5:** Relationships between QSM values in cortical regions and multi‐domain cognitive scores in T2DM group: multiple linear regression analysis.
**Table S7:** Relationships between QSM values in limbic system subregions and multi‐domain cognitive scores in T2DM group: multiple linear regression analysis.
**Table S8:** Moderating effect of HbA1c on the association between QSM values in cortical regions and cognitive scores.
**Table S9:** Moderating effect of HbA1c on the association between limbic subregional QSM values and cognitive scores.
**Table S10:** SVR prediction performance for TMT‐A from limbic subregional QSM values in the T2DM group.
**Table S11:** Demographic, clinical, and cognitive characteristics of study participants in the PSM cohort.
**Table S12:** QSM values for cortex ROIs in both T2DM and HC groups in the PSM cohort.
**Table S13:** QSM values for limbic subregional ROIs in both T2DM and HC groups in the PSM cohort.
**Table S14:** Relationship between cortical QSM values and glycemic metabolic profiles in all participants within a PSM cohort.
**Table S15:** Relationship between limbic subregional QSM values and glycemic metabolic profiles in all participants in the PSM cohort.
**Table S16:** Relationships between QSM values in cortical regions and multi‐domain cognitive scores in all participants: multiple linear regression analysis in the PSM cohort.
**Table S17:** Relationships between QSM values in cortical regions and multi‐domain cognitive scores in T2DM group: multiple linear regression analysis in the PSM cohort.
**Table S18:** Relationships between QSM values in limbic system subregions and multi‐domain cognitive scores in all participants: multiple linear regression analysis in the PSM cohort.
**Table S19:** Relationships between QSM values in limbic system subregions and multi‐domain cognitive scores in T2DM group: multiple linear regression analysis in the PSM cohort.
**Table S20:** Moderating effect of HbA1c on the association between QSM values in cortical and limbic subregions and cognitive scores in the PSM cohort.
**Table S21:** Associations between cortical and limbic subregion QSM values and TMT‐A scores at different HbA1c levels in the PSM cohort.
**Table S22:** SVR prediction performance for TMT‐A from limbic subregional QSM values in the T2DM group in PSM cohort.
**Table S23:** Between‐group comparisons of limbic subregional QSM values in the PSM‐matched cohort after additional adjustment for DBP, HDL‐C, and hypertension.

## Data Availability

The data that support the findings of this study are available on request from the corresponding author. The data are not publicly available due to privacy or ethical restrictions.
