# Supplementary material for: Limbic‐Predominant Cortical Magnetic Susceptibility Alterations Are Associated With Glycemic Dysregulation and Cognitive Performance in Type 2 Diabetes
Source: CNS Neurosci Ther. 2026 Sep 25;32(9):e71181. doi: 10.1002/cns.71181 (PMC13613945; doi:10.1002/cns.71181)
Supplement: Supplementary file 1 [file CNS-32-e71181-s001.docx]

**Appendix E1：Detailed Exclusion Criteria**

The exclusion criteria for all participants were as follows: (i) history of neurological or psychiatric disorders, including stroke, traumatic brain injury, Parkinson’s disease, history of alcohol abuse, or any other neurological/psychiatric conditions identified through clinical assessment and medical history review; (ii) systemic or other medical conditions, including major medical illnesses (e.g., cancer, anemia, chronic kidney disease), autoimmune diseases, other endocrine disorders, or severe visual or hearing impairment; and (iii) contraindications for MRI scanning, such as claustrophobia, pregnancy, or the presence of metallic implants (e.g., cardiac pacemakers, coronary stents).

**Appendix E2：MRI Data Acquisition**

All participants underwent scanning on a 3.0-T MRI system (MAGNETOM Prisma, Siemens Healthcare, Erlangen, Germany). The data acquisition protocols included:

(1) **Structural Imaging**: High-resolution structural images were acquired using a 3D T1-weighted **Magnetization-Prepared Rapid Gradient-Echo (MPRAGE)** with the following parameters: repetition time (TR) = 2530 ms; echo time (TE) = 2.98 ms; flip angle (FA) = 7°; field of view (FOV) = 256 mm × 256 mm; acquisition matrix = 256 × 256; slice thickness = 1 mm; voxel size = 1.0 × 1.0 × 1.0 mm³; and 192 sagittal slices.

(2) **Quantitative Susceptibility Mapping** (QSM): A 3D multi-echo gradient-recalled echo (mGRE) sequence was employed with the following parameters: TR = 55 ms; 8 echoes (TEs = 6.15, 11.7, 17.25, 22.8, 28.35, 33.9, 39.45, and 45 ms); FA = 15°; FOV = 220 mm × 220 mm; acquisition matrix = 256 × 256; slice thickness = 2 mm; voxel size = 0.86 × 0.86 × 2.0 mm³; and 64 contiguous axial slices.

**Appendix E3：Anatomical parcellation and registration**

Image quality control. Prior to processing, all T1-weighted MPRAGE and QSM images were visually inspected to identify excessive motion artifacts, insufficient signal-to-noise ratio, geometric distortion, or incidental brain pathology. QSM images were additionally screened for severe streaking artifacts or signal dropout.

Automated brain structural analysis and segmentation of the 3D T1-weighted MPRAGE images were performed using FreeSurfer (1) version 6.0 (<http://surfer.nmr.mgh.harvard.edu/>). The segmentation results underwent rigorous quality control via visual inspection by a neuroimaging researcher with 10 years of experience. Specifically, inspection included (i) the completeness of skull stripping, (ii) the accuracy of white matter and pial surface boundaries, and (iii) the anatomical plausibility of cortical parcellation labels based on the Desikan-Killiany atlas, with particular attention to the cingulate, parahippocampal, and entorhinal regions. Cortical parcellations were aggregated into lobar regions to generate regions of interest (ROI) masks for the bilateral frontal, parietal, occipital, temporal, insular, and limbic cortices, totaling 12 distinct lobar regions.

Multimodal image registration was conducted using the Advanced Normalization Tools (2) (ANTs, [http://stnava.github.io/ ANTs/](https://github.com/antsx/ants)). Specifically, the T1-weighted images were first registered to the magnitude image of the first echo (TE = 6.15 ms) in the GRE sequence. Subsequently, the QSM images were transformed from GRE space to T1w space using the inverse transformation matrix, ensuring precise spatial alignment between the quantitative QSM values and the T1w-based anatomical ROIs. A rigid transformation was employed during registration to preserve image geometry. Registration quality was verified by overlaying the co-registered QSM maps onto the T1w images for each subject, confirming alignment of cortical boundaries and anatomical landmarks.

A detailed subject inclusion and image quality-control flowchart and representative examples of image quality control are provided in Supplementary Figures S1 and S2, respectively.

**Appendix E4：Sensitivity Analysis Results**

Consistent with the primary analysis, T2DM patients in the matched cohort still showed a higher prevalence of hypertension, elevated glycemic metabolic indicators (e.g., FPG, HbA1c), and higher lipid levels, all of which were statistically significant. In terms of cognitive function assessment (Table S11), T2DM patients in the matched cohort continued to exhibit significant cognitive decline in specific cognitive domains, such as the AVLT reflecting memory function and the TMT-A reflecting executive function. These findings remained consistent with the primary analysis.

Regarding the QSM analysis of brain iron deposition, differences in limbic lobe cortical gray matter QSM values between the T2DM and HC groups persisted in the matched cohort (Table S12). Further sub-regional analysis indicated that these differences were primarily driven by the cingulate cortex (Left: *t* = 2.795, *P* = 0.006, *P_FDR_* = 0.019; Right: *t* = 2.027, *P* = 0.044, *P_FDR_* = 0.079) and the right parahippocampal gyrus (*t* = 2.756, *P* = 0.006, *P_FDR_* = 0.019). Details are provided in Figure S3 and Table S13.

Furthermore, within the entire matched cohort, we continued to confirm a significant positive correlation between cortical gray matter QSM values and glycemic metabolic indicators (Figure S4 and Tables S14-S15). For instance, FPG was significantly positively correlated with QSM values in the bilateral limbic cortex (left: *r* = 0.189, *P* = 0.006, *P_FDR_* = 0.036; right: *r* = 0.2, *P* = 0.004, *P_FDR_* = 0.036). In the sub-regional analysis, the bilateral cingulate gyrus (left: *r* = 0.245, *P <* 0.001, *P_FDR_* = 0.003; right: *r* = 0.223, *P* = 0.001, *P_FDR_* = 0.005) remained the primary driving factor, and these associations stayed significant after FDR correction.

In investigations exploring the association between cortical QSM values and cognitive domains, within the entire PSM cohort, we still observed a significant negative correlation between the limbic cortex (left: *β* = -0.012, *P* = 0.034; right: *β* = -0.014, *P* = 0.011) and its cingulate cortex (left: *β* = -0.014, *P* = 0.017; right: *β* = -0.015, *P* = 0.011) and the CDT scores. Consistent negative correlations were also observed in the T2DM subgroup. However, several significant correlations between cortical gray matter QSM values and TMT-A, which were found in the full cohort in the primary analysis, were not entirely replicated in the sensitivity analysis. This might be attributed to the reduced sample size after PSM, leading to decreased statistical power. Nevertheless, in the T2DM subgroup, a significant correlation still existed between elevated right frontal cortex QSM values (reflecting higher iron load) and slower TMT-A performance (*β* = 0.555, *P* = 0.039). Similarly, the significant correlation between increased right entorhinal cortex QSM values and lower MMSE scores (*β* = -0.014, *P* = 0.033) in the T2DM group also remained. The findings are detailed in Figure S5 and Tables S16-19.

In the PSM-matched cohort, moderation analyses similarly revealed that HbA1c moderated the association between limbic cortical QSM values and cognitive performance after controlling for diagnostic group effects (Figure S6, Table S20). Specifically, HbA1c moderated the association between right limbic cortex QSM values and CDT scores (β = -0.010, 95% CI [-0.016, -0.003], *P* = 0.004), right parahippocampal gyrus QSM values and MoCA scores (β = -0.032, 95% CI [-0.054, -0.010], *P* = 0.005), and right cingulate cortex QSM values and CDT scores (β = -0.011, 95% CI [-0.019, -0.004], *P* = 0.003). Simple slope analyses confirmed the pattern observed in the primary analysis: at high HbA1c levels (+1 SD), higher QSM values were associated with poorer cognitive scores (all *P* < 0.01), whereas no such associations were observed at low HbA1c levels (all *P* > 0.50; Table S21).

Similarly, within the PSM-matched cohort, SVR modeling showed that QSM values across limbic subregions predicted TMT-A performance in the T2DM group (Figure S7 and Table S22). The predictive associations were observed for the bilateral cingulate cortices (left: r = 0.558, R² = 0.224; right: r = 0.576, R² = 0.228), the bilateral parahippocampal gyri (left: *r* = 0.496, R² = 0.121; right: *r* = 0.551, R² = 0.220) and bilateral entorhinal cortices (left: *r* = 0.573, R² = 0.196; right: *r* = 0.589, R² = 0.266), with all models surviving FDR correction (all *P_FDR_* < 0.001). Bootstrap 95% confidence intervals for all performance metrics are reported in Table S22.

**Appendix E5: Additional adjustment for residual imbalance after PSM**

After PSM, residual imbalances (|SMD| > 0.2) remained in several variables. FPG and HbA1c are diagnostic criteria for T2DM and were not adjusted for. DBP, HDL-C, and hypertension, which reflect the cardiovascular comorbidity profile commonly associated with T2DM, were included as additional covariates in a further sensitivity analysis of the between-group comparisons for the limbic subregions — the primary regions of interest in this study (Figure S8 and Tables S23). The results were largely consistent with the main analysis.

**References**

1.	B. Fischl *et al.*, Whole brain segmentation: automated labeling of neuroanatomical structures in the human brain. *Neuron* **33**, 341-355 (2002).

2.	N. J. Tustison *et al.*, The ANTsX ecosystem for quantitative biological and medical imaging. *Sci Rep* **11**, 9068 (2021).

**Figure S1. Subject inclusion and image quality control flowchart.** Overview of the screening and exclusion process for image quality control, FreeSurfer cortical segmentation, and QSM-to-T1w registration. Numbers on the right at each stage indicate the number of subjects excluded. The final analytic sample comprised 145 patients with T2DM and 182 healthy controls.

**Supplementary Figures**

**Figure S2. Representative quality control examples. (A)** FreeSurfer cortical segmentation quality, with pial surfaces (red) and white matter surfaces (yellow) overlaid on the T1-weighted images in the axial plane. **(B)** QSM-to-T1w registration accuracy, with the co-registered QSM maps overlaid on the T1-weighted images. **(C)** Cortical parcellation labels based on the Desikan-Killiany atlas overlaid on the T1-weighted images.

**Figure S3. Violin plots illustrating differences in QSM values across limbic subregions between the T2DM and HC groups in PSM cohort**

**Figure S4.** **Correlations between cortical QSM values and glucose metabolism indicators in PSM cohort. (A)** Correlations between limbic cortical gray matter QSM values and glucose metabolism indicators. **(B)** Correlations between cingulate gray matter QSM values and glucose metabolism indicators.

**Figure S5. Results of multiple linear regression analysis in PSM cohort.** Relationships between QSM values in cortical regions and multi-domain cognitive scores. Values in parentheses represent 95% confidence intervals (CI). Statistical significance is defined as *P* < 0.05. For the complete list of model coefficients, please refer to Tables S16-19.

**Figure S6. Simple slope analysis plots in PSM cohort.** The association between QSM values and cognitive scores is plotted at low (−1 SD), mean, and high (+1 SD) levels of HbA1c. Detailed results of the moderation analyses and simple slope analyses are provided in Tables 20-21.

**Figure S7. Prediction of TMT-A scores in patients with T2DM in in PSM cohort.** Scatter plots show the linear relationship between the predicted and measured clinical TMT-A scores, with the Spearman correlation coefficient (*r*), coefficient of determination (R²), and their corresponding 95% confidence intervals reported to assess predictive performance.

**Figure S8. Between-group comparisons of limbic subregional QSM values in the PSM-matched cohort after additional adjustment for DBP, HDL-C, and hypertension.** Violin plots with overlaid box plots and individual data points. P values are from ANCOVA adjusting for age, sex, education, DBP, HDL-C, and hypertension.

**Supplementary Tables**

**Table S1: QSM values for** **limbic cortex ROIs in both T2DM and HC groups**

| **Region** | **T2DM** | **HC** | **statistic** | ***P* value** | ***P_FDR_*** |
| --- | --- | --- | --- | --- | --- |
| Left cingulate cortex | -1.32 (-8.11, 4.30) | -8.52 (-15.00, 0.35) | 2.849 | **0.005** | **0.019^*^** |
| Right cingulate cortex | -2.53 (-9.07, 3.87) | -6.84 (-13.80, 0.54) | 1.923 | 0.055 | 0.924 |
| Left entorhinal cortex | -16.38 (-36.12, 1.50) | -14.99 (-35.96, 3.26) | -0.096 | 0.924 | 0.217 |
| Right entorhinal cortex | -19.09 (-35.13, -1.33) | -14.72 (-34.76, 0.02) | -0.756 | 0.45 | 0.111 |
| Left parahippocampal | 10.74 (1.94, 18.13) | 8.76 (1.04, 14.43) | 1.461 | 0.145 | 0.54 |
| Right parahippocampal | 14.63 (8.50, 20.75) | 10.49 (5.01, 16.68) | 2.747 | **0.006** | **0.019^*^** |

***Note.*** Significant results (*P* < 0.05) are reported in bold. ^*^ indicates *P_FDR_* < 0.05.

**Table S2: Relationship between cortical QSM values and glycemic metabolic profiles in all participants**

|  | FPG | | | HbA1c | | | FINS | | |
| --- | --- | --- | --- | --- | --- | --- | --- | --- | --- |
|  | ***r*** | ***P*** | ***P_FDR_*** | ***r*** | ***P*** | ***P_FDR_*** | ***r*** | ***P*** | ***P_FDR_*** |
| Left frontal cortex | 0.091 | 0.116 | 0.327 | 0.094 | 0.102 | 0.306 | 0.091 | 0.119 | 0.237 |
| Right frontal cortex | 0.077 | 0.181 | 0.353 | 0.064 | 0.268 | 0.459 | 0.084 | 0.151 | 0.259 |
| Left parietal cortex | 0.047 | 0.412 | 0.494 | 0.044 | 0.447 | 0.671 | 0.067 | 0.250 | 0.337 |
| Right parietal cortex | 0.001 | 0.985 | 0.985 | 0.017 | 0.766 | 0.836 | 0.056 | 0.337 | 0.337 |
| Left temporal cortex | 0.034 | 0.558 | 0.609 | 0.030 | 0.602 | 0.802 | 0.064 | 0.277 | 0.337 |
| Right temporal cortex | 0.068 | 0.236 | 0.353 | 0.074 | 0.196 | 0.420 | 0.058 | 0.317 | 0.337 |
| Left occipital cortex | 0.073 | 0.206 | 0.353 | 0.072 | 0.210 | 0.420 | **0.118** | **0.043** | 0.109 |
| Right occipital cortex | 0.128 | 0.026 | 0.104 | 0.094 | 0.101 | 0.306 | **0.137** | **0.019** | 0.109 |
| Left insular cortex | 0.086 | 0.136 | 0.327 | 0.018 | 0.752 | 0.836 | **0.147** | **0.012** | 0.109 |
| Right insular cortex | 0.054 | 0.349 | 0.465 | 0.004 | 0.946 | 0.946 | **0.117** | **0.045** | 0.109 |
| Left limbic cortex | **0.170** | **0.003** | **0.018^*^** | **0.131** | **0.022** | 0.268 | **0.125** | **0.032** | 0.109 |
| Right limbic cortex | **0.178** | **0.002** | **0.018^*^** | **0.114** | **0.046** | 0.277 | 0.063 | 0.283 | 0.337 |

***Note.*** Significant results (*P* < 0.05) are reported in bold. ^*^ Indicates *P_FDR_* < 0.05.

**Table S3: Partial correlations between QSM values and glycemic metabolic indices in all participants.**

|  | FPG | | | HbA1c | | | FINS | | |
| --- | --- | --- | --- | --- | --- | --- | --- | --- | --- |
|  | ***r*** | ***P*** | ***P_FDR_*** | ***r*** | ***P*** | ***P_FDR_*** | ***r*** | ***P*** | ***P_FDR_*** |
| Left cingulate cortex | **0.219** | **<.001** | **0.001^*^** | **0.170** | **0.003** | **0.018^*^** | **0.128** | **0.027** | 0.082 |
| Right cingulate cortex | **0.197** | **0.001** | **0.002^*^** | **0.134** | **0.019** | 0.058 | 0.084 | 0.151 | 0.302 |
| Left entorhinal cortex | 0.001 | 0.981 | 0.981 | -0.018 | 0.760 | 0.912 | -0.014 | 0.812 | 0.812 |
| Right entorhinal cortex | 0.054 | 0.353 | 0.550 | 0.000 | 0.998 | 0.998 | -0.028 | 0.629 | 0.755 |
| Left parahippocampal | 0.049 | 0.392 | 0.550 | 0.051 | 0.373 | 0.559 | 0.134 | 0.022 | 0.082 |
| Right parahippocampal | 0.043 | 0.458 | 0.550 | 0.083 | 0.149 | 0.297 | 0.049 | 0.404 | 0.606 |

***Note.*** Significant results (*P* < 0.05) are reported in bold. ^*^ Indicates *P_FDR_* < 0.05

**Table S4: Relationships between QSM values in cortical regions and multi-domain cognitive scores in all participants: multiple linear regression analysis**

| **Cognitive**  **Function** | **Left frontal cortex** | | | | **Right frontal cortex** | | | | **Left parietal cortex** | | | | **Right parietal cortex** | | | |
| --- | --- | --- | --- | --- | --- | --- | --- | --- | --- | --- | --- | --- | --- | --- | --- | --- |
|  | β | 95% CI | | *P* | β | 95% CI | | *P* | β | 95% CI | | *P* | β | 95% CI | | *P* |
| MoCA | -0.024 | -0.057 | 0.009 | 0.156 | -0.026 | -0.06 | 0.008 | 0.128 | -0.017 | -0.051 | 0.017 | 0.333 | -0.024 | -0.06 | 0.012 | 0.188 |
| MMSE | 0.003 | -0.026 | 0.032 | 0.856 | -0.004 | -0.033 | 0.026 | 0.8 | -0.007 | -0.037 | 0.023 | 0.651 | -0.004 | -0.035 | 0.028 | 0.822 |
| AVLT-immediate | -0.024 | -0.084 | 0.037 | 0.442 | -0.021 | -0.083 | 0.04 | 0.5 | -0.001 | -0.064 | 0.062 | 0.979 | -0.012 | -0.078 | 0.054 | 0.717 |
| AVLT-5min | -0.014 | -0.042 | 0.014 | 0.326 | -0.018 | -0.046 | 0.01 | 0.213 | -0.01 | -0.039 | 0.019 | 0.483 | -0.009 | -0.04 | 0.021 | 0.558 |
| AVLT-delay | -0.016 | -0.048 | 0.015 | 0.309 | -0.024 | -0.057 | 0.008 | 0.135 | -0.007 | -0.04 | 0.026 | 0.667 | -0.011 | -0.046 | 0.024 | 0.537 |
| AVLT-recall | **-0.025** | **-0.043** | **-0.006** | **0.009** | **-0.022** | **-0.04** | **-0.003** | **0.023** | -0.011 | -0.03 | 0.008 | 0.254 | -0.017 | -0.037 | 0.003 | 0.099 |
| TMT-A | **0.285** | **0.028** | **0.541** | **0.03** | **0.28** | **0.019** | **0.541** | **0.035** | **0.292** | **0.026** | **0.558** | **0.032** | **0.376** | **0.097** | **0.656** | **0.009** |
| DST-forward | 0.021 | -0.007 | 0.048 | 0.139 | 0.018 | -0.009 | 0.046 | 0.194 | 0.022 | -0.007 | 0.05 | 0.136 | 0.026 | -0.003 | 0.056 | 0.082 |
| DST-backward | -0.003 | -0.021 | 0.014 | 0.709 | -0.004 | -0.022 | 0.014 | 0.631 | -0.006 | -0.025 | 0.012 | 0.486 | -0.01 | -0.029 | 0.01 | 0.323 |
| GPT-R | 0.246 | -0.076 | 0.569 | 0.133 | 0.217 | -0.111 | 0.545 | 0.193 | 0.126 | -0.209 | 0.461 | 0.46 | 0.208 | -0.145 | 0.56 | 0.247 |
| GPT-L | 0.159 | -0.166 | 0.484 | 0.338 | 0.157 | -0.173 | 0.487 | 0.35 | 0.109 | -0.227 | 0.445 | 0.525 | 0.166 | -0.189 | 0.52 | 0.358 |
| CDT | -0.006 | -0.016 | 0.005 | 0.277 | -0.009 | -0.02 | 0.001 | 0.088 | -0.008 | -0.019 | 0.003 | 0.145 | -0.008 | -0.019 | 0.003 | 0.171 |
| **Cognitive**  **Function** | **Left temporal cortex** | | | | **Right temporal cortex** | | | | **Left occipital cortex** | | | | **Right occipital cortex** | | | |
|  | β | 95% CI | | *P* | β | 95% CI | | *P* | β | 95% CI | | *P* | β | 95% CI | | *P* |
| MoCA | -0.015 | -0.047 | 0.018 | 0.372 | -0.01 | -0.041 | 0.022 | 0.552 | -0.016 | -0.046 | 0.014 | 0.304 | -0.006 | -0.036 | 0.023 | 0.67 |
| MMSE | -0.004 | -0.032 | 0.025 | 0.792 | 0.004 | -0.023 | 0.032 | 0.758 | -0.001 | -0.027 | 0.025 | 0.934 | -0.001 | -0.027 | 0.024 | 0.929 |
| AVLT-immediate | -0.008 | -0.067 | 0.051 | 0.789 | 0 | -0.058 | 0.058 | 0.999 | 0.01 | -0.045 | 0.065 | 0.723 | 0.017 | -0.037 | 0.071 | 0.535 |
| AVLT-5min | -0.008 | -0.035 | 0.02 | 0.578 | -0.002 | -0.028 | 0.025 | 0.895 | 0.004 | -0.021 | 0.03 | 0.738 | 0.001 | -0.024 | 0.026 | 0.951 |
| AVLT-delay | -0.004 | -0.035 | 0.027 | 0.796 | -0.004 | -0.034 | 0.026 | 0.785 | 0.004 | -0.025 | 0.033 | 0.792 | 0.002 | -0.026 | 0.03 | 0.893 |
| AVLT-recall | -0.017 | -0.035 | 0.001 | 0.064 | -0.014 | -0.031 | 0.004 | 0.123 | -0.011 | -0.028 | 0.005 | 0.182 | -0.009 | -0.025 | 0.007 | 0.285 |
| TMT-A | 0.152 | -0.102 | 0.405 | 0.24 | 0.138 | -0.107 | 0.384 | 0.269 | 0.217 | -0.016 | 0.451 | 0.068 | 0.143 | -0.085 | 0.372 | 0.219 |
| DST-forward | 0.014 | -0.013 | 0.041 | 0.295 | 0.013 | -0.013 | 0.039 | 0.32 | 0.019 | -0.006 | 0.044 | 0.141 | 0.016 | -0.008 | 0.04 | 0.198 |
| DST-backward | -0.011 | -0.028 | 0.007 | 0.231 | -0.007 | -0.024 | 0.01 | 0.418 | -0.004 | -0.02 | 0.012 | 0.603 | -0.006 | -0.022 | 0.009 | 0.435 |
| GPT-R | 0.164 | -0.153 | 0.481 | 0.309 | 0.147 | -0.16 | 0.454 | 0.348 | 0.216 | -0.077 | 0.509 | 0.148 | 0.144 | -0.141 | 0.43 | 0.32 |
| GPT-L | 0.119 | -0.2 | 0.439 | 0.463 | 0.127 | -0.183 | 0.436 | 0.421 | 0.086 | -0.209 | 0.38 | 0.568 | 0.1 | -0.187 | 0.386 | 0.494 |
| CDT | -0.005 | -0.015 | 0.005 | 0.337 | -0.006 | -0.016 | 0.004 | 0.267 | -0.005 | -0.015 | 0.004 | 0.263 | -0.005 | -0.014 | 0.004 | 0.298 |
| **Cognitive**  **Function** | **Left insular cortex** | | | | **Right insular cortex** | | | | **Left limbic cortex** | | | | **Right limbic cortex** | | | |
|  | β | 95% CI | | *P* | β | 95% CI | | *P* | β | 95% CI | | *P* | β | 95% CI | | P |
| MoCA | -0.009 | -0.039 | 0.02 | 0.531 | -0.021 | -0.052 | 0.01 | 0.176 | -0.016 | -0.043 | 0.01 | 0.233 | -0.013 | -0.041 | 0.014 | 0.346 |
| MMSE | 0.002 | -0.024 | 0.028 | 0.885 | -0.003 | -0.03 | 0.024 | 0.823 | -0.015 | -0.038 | 0.008 | 0.191 | -0.014 | -0.037 | 0.01 | 0.26 |
| AVLT-immediate | 0.021 | -0.033 | 0.076 | 0.438 | 0.009 | -0.048 | 0.065 | 0.767 | -0.021 | -0.07 | 0.027 | 0.391 | -0.005 | -0.055 | 0.045 | 0.844 |
| AVLT-5min | 0.002 | -0.022 | 0.027 | 0.845 | -0.004 | -0.03 | 0.022 | 0.742 | -0.014 | -0.036 | 0.009 | 0.227 | -0.014 | -0.037 | 0.009 | 0.247 |
| AVLT-delay | 0.004 | -0.025 | 0.032 | 0.797 | -0.008 | -0.038 | 0.021 | 0.573 | -0.008 | -0.033 | 0.018 | 0.553 | -0.01 | -0.036 | 0.016 | 0.463 |
| AVLT-recall | -0.011 | -0.028 | 0.005 | 0.171 | -0.007 | -0.025 | 0.01 | 0.387 | -0.012 | -0.027 | 0.002 | 0.096 | -0.011 | -0.026 | 0.004 | 0.154 |
| TMT-A | 0.142 | -0.087 | 0.371 | 0.224 | 0.177 | -0.062 | 0.415 | 0.146 | **0.283** | **0.079** | **0.487** | **0.007** | **0.302** | **0.093** | **0.512** | **0.005** |
| DST-forward | 0.02 | -0.004 | 0.045 | 0.1 | 0.016 | -0.01 | 0.041 | 0.229 | 0.009 | -0.012 | 0.031 | 0.395 | 0.014 | -0.008 | 0.037 | 0.218 |
| DST-backward | -0.001 | -0.017 | 0.015 | 0.915 | -0.003 | -0.02 | 0.013 | 0.678 | -0.003 | -0.017 | 0.011 | 0.685 | -0.006 | -0.02 | 0.009 | 0.422 |
| GPT-R | 0.088 | -0.2 | 0.375 | 0.55 | -0.002 | -0.302 | 0.299 | 0.991 | **0.275** | **0.019** | **0.53** | **0.035** | 0.249 | -0.015 | 0.513 | 0.065 |
| GPT-L | 0.014 | -0.276 | 0.304 | 0.925 | -0.032 | -0.333 | 0.27 | 0.837 | 0.211 | -0.046 | 0.469 | 0.107 | 0.183 | -0.083 | 0.449 | 0.177 |
| CDT | -0.007 | -0.017 | 0.002 | 0.132 | -0.009 | -0.018 | 0.001 | 0.081 | **-0.008** | **-0.017** | **0** | **0.048** | **-0.01** | **-0.019** | **-0.001** | **0.022** |

***Note.*** Significant results (*P* < 0.05) are reported in bold.

**Table S5: Relationships between** **QSM values in cortical regions and multi-domain cognitive scores in T2DM group: multiple linear regression analysis**

| **Cognitive**  **Function** | **Left frontal cortex** | | | | **Right frontal cortex** | | | | **Left parietal cortex** | | | | **Right parietal cortex** | | | |
| --- | --- | --- | --- | --- | --- | --- | --- | --- | --- | --- | --- | --- | --- | --- | --- | --- |
|  | β | 95% CI | | *P* | β | 95% CI | | *P* | β | 95% CI | | *P* | β | 95% CI | | *P* |
| MoCA | -0.018 | -0.069 | 0.034 | 0.497 | -0.031 | -0.085 | 0.022 | 0.252 | -0.017 | -0.072 | 0.038 | 0.539 | -0.016 | -0.074 | 0.042 | 0.586 |
| MMSE | -0.005 | -0.041 | 0.030 | 0.764 | -0.006 | -0.043 | 0.031 | 0.756 | -0.001 | -0.039 | 0.037 | 0.952 | 0.002 | -0.037 | 0.042 | 0.907 |
| AVLT-immediate | -0.035 | -0.128 | 0.059 | 0.466 | -0.046 | -0.144 | 0.052 | 0.355 | 0.008 | -0.092 | 0.108 | 0.880 | -0.024 | -0.130 | 0.081 | 0.649 |
| AVLT-5min | -0.009 | -0.055 | 0.036 | 0.691 | -0.020 | -0.068 | 0.027 | 0.404 | 0.001 | -0.047 | 0.049 | 0.964 | -0.005 | -0.057 | 0.046 | 0.833 |
| AVLT-delay | -0.011 | -0.063 | 0.041 | 0.679 | -0.027 | -0.082 | 0.027 | 0.321 | 0.006 | -0.049 | 0.062 | 0.820 | -0.002 | -0.061 | 0.056 | 0.937 |
| AVLT-recall | -0.033 | -0.067 | 0.001 | 0.057 | -0.036 | **-0.071** | **0.000** | **0.049** | -0.016 | -0.053 | 0.021 | 0.395 | -0.030 | -0.069 | 0.008 | 0.124 |
| TMT-A | 0.312 | -0.036 | 0.659 | 0.078 | 0.439 | **0.079** | **0.800** | **0.017** | 0.337 | -0.033 | 0.707 | 0.074 | 0.297 | -0.097 | 0.691 | 0.138 |
| DST-forward | 0.014 | -0.043 | 0.071 | 0.625 | 0.009 | -0.050 | 0.069 | 0.761 | 0.016 | -0.045 | 0.077 | 0.606 | 0.021 | -0.042 | 0.085 | 0.510 |
| DST-backward | -0.011 | -0.040 | 0.017 | 0.423 | -0.009 | -0.038 | 0.021 | 0.568 | -0.013 | -0.043 | 0.018 | 0.411 | -0.013 | -0.044 | 0.019 | 0.433 |
| GPT-R | -0.039 | -0.590 | 0.512 | 0.889 | -0.020 | -0.601 | 0.561 | 0.946 | -0.190 | -0.777 | 0.397 | 0.522 | -0.185 | -0.807 | 0.437 | 0.557 |
| GPT-L | -0.037 | -0.527 | 0.453 | 0.883 | 0.098 | -0.417 | 0.613 | 0.708 | 0.053 | -0.465 | 0.571 | 0.840 | 0.045 | -0.506 | 0.596 | 0.873 |
| CDT | -0.006 | -0.025 | 0.013 | 0.516 | -0.013 | -0.033 | 0.007 | 0.188 | -0.009 | -0.029 | 0.011 | 0.384 | -0.010 | -0.031 | 0.011 | 0.363 |
| **Cognitive**  **Function** | **Left temporal cortex** | | | | **Right temporal cortex** | | | | **Left occipital cortex** | | | | **Right occipital cortex** | | | |
|  | β | 95% CI | | *P* | β | 95% CI | | *P* | β | 95% CI | | *P* | β | 95% CI | | *P* |
| MoCA | -0.019 | -0.074 | 0.036 | 0.493 | -0.012 | -0.069 | 0.046 | 0.689 | -0.024 | -0.071 | 0.023 | 0.317 | -0.014 | -0.061 | 0.033 | 0.559 |
| MMSE | 0.010 | -0.028 | 0.048 | 0.597 | 0.019 | -0.021 | 0.059 | 0.340 | 0.003 | -0.030 | 0.035 | 0.871 | -0.004 | -0.036 | 0.029 | 0.826 |
| AVLT-immediate | -0.017 | -0.117 | 0.082 | 0.731 | 0.000 | -0.105 | 0.106 | 0.999 | -0.009 | -0.095 | 0.078 | 0.846 | -0.004 | -0.090 | 0.082 | 0.921 |
| AVLT-5min | -0.004 | -0.052 | 0.044 | 0.876 | 0.007 | -0.044 | 0.058 | 0.790 | 0.007 | -0.035 | 0.048 | 0.756 | 0.003 | -0.038 | 0.045 | 0.881 |
| AVLT-delay | -0.006 | -0.062 | 0.049 | 0.816 | -0.003 | -0.061 | 0.056 | 0.933 | 0.008 | -0.040 | 0.056 | 0.742 | 0.000 | -0.047 | 0.048 | 0.985 |
| AVLT-recall | -0.031 | -0.067 | 0.005 | 0.089 | -0.035 | -0.073 | 0.003 | 0.069 | -0.014 | -0.046 | 0.017 | 0.372 | -0.018 | -0.049 | 0.013 | 0.256 |
| TMT-A | 0.297 | -0.078 | 0.672 | 0.119 | 0.121 | -0.276 | 0.519 | 0.547 | 0.278 | -0.042 | 0.597 | 0.088 | 0.219 | -0.098 | 0.536 | 0.174 |
| DST-forward | 0.016 | -0.045 | 0.077 | 0.601 | 0.017 | -0.047 | 0.081 | 0.599 | 0.013 | -0.039 | 0.066 | 0.611 | 0.005 | -0.047 | 0.056 | 0.856 |
| DST-backward | -0.014 | -0.044 | 0.017 | 0.374 | -0.008 | -0.040 | 0.024 | 0.626 | -0.011 | -0.037 | 0.014 | 0.384 | -0.013 | -0.038 | 0.013 | 0.320 |
| GPT-R | -0.140 | -0.731 | 0.451 | 0.640 | -0.269 | -0.891 | 0.353 | 0.393 | 0.062 | -0.444 | 0.568 | 0.808 | -0.078 | -0.577 | 0.421 | 0.758 |
| GPT-L | -0.090 | -0.614 | 0.435 | 0.736 | -0.164 | -0.723 | 0.394 | 0.561 | -0.012 | -0.458 | 0.434 | 0.958 | -0.067 | -0.506 | 0.373 | 0.764 |
| CDT | -0.008 | -0.028 | 0.012 | 0.425 | -0.010 | -0.031 | 0.012 | 0.375 | -0.009 | -0.027 | 0.008 | 0.286 | -0.013 | -0.030 | 0.004 | 0.139 |
| **Cognitive**  **Function** | **Left insular cortex** | | | | **Right insular cortex** | | | | **Left limbic cortex** | | | | **Right limbic cortex** | | | |
|  | β | 95% CI | | *P* | β | 95% CI | | ***P*** | β | 95% CI | | *P* | β | 95% CI | | *P* |
| MoCA | 0.005 | -0.041 | 0.051 | 0.830 | -0.018 | -0.064 | 0.029 | 0.446 | -0.012 | -0.056 | 0.031 | 0.572 | -0.023 | -0.069 | 0.024 | 0.338 |
| MMSE | 0.003 | -0.029 | 0.034 | 0.868 | 0.000 | -0.031 | 0.032 | 0.984 | -0.011 | -0.041 | 0.018 | 0.447 | -0.022 | -0.053 | 0.009 | 0.162 |
| AVLT-immediate | 0.027 | -0.057 | 0.111 | 0.525 | 0.021 | -0.064 | 0.106 | 0.628 | -0.021 | -0.101 | 0.059 | 0.605 | -0.020 | -0.105 | 0.065 | 0.646 |
| AVLT-5min | 0.018 | -0.023 | 0.058 | 0.393 | 0.006 | -0.035 | 0.047 | 0.784 | -0.014 | -0.053 | 0.024 | 0.467 | -0.024 | -0.064 | 0.017 | 0.256 |
| AVLT-delay | 0.025 | -0.021 | 0.072 | 0.282 | 0.010 | -0.037 | 0.057 | 0.662 | 0.002 | -0.042 | 0.046 | 0.917 | -0.009 | -0.056 | 0.038 | 0.702 |
| AVLT-recall | -0.018 | -0.048 | 0.012 | 0.232 | -0.009 | -0.039 | 0.021 | 0.548 | -0.025 | -0.053 | 0.003 | 0.081 | -0.024 | -0.054 | 0.005 | 0.107 |
| TMT-A | 0.210 | -0.100 | 0.520 | 0.183 | 0.233 | -0.077 | 0.542 | 0.139 | 0.230 | -0.060 | 0.520 | 0.118 | **0.351** | **0.045** | **0.657** | **0.025** |
| DST-forward | 0.018 | -0.033 | 0.068 | 0.486 | 0.019 | -0.032 | 0.069 | 0.468 | 0.003 | -0.045 | 0.050 | 0.908 | 0.015 | -0.036 | 0.065 | 0.572 |
| DST-backward | -0.003 | -0.028 | 0.022 | 0.821 | -0.010 | -0.035 | 0.016 | 0.454 | -0.008 | -0.031 | 0.016 | 0.514 | -0.013 | -0.038 | 0.012 | 0.309 |
| GPT-R | -0.138 | -0.629 | 0.354 | 0.579 | -0.199 | -0.693 | 0.296 | 0.428 | 0.094 | -0.364 | 0.551 | 0.686 | 0.189 | -0.299 | 0.676 | 0.445 |
| GPT-L | -0.062 | -0.501 | 0.376 | 0.779 | -0.077 | -0.514 | 0.360 | 0.728 | 0.058 | -0.345 | 0.462 | 0.775 | 0.104 | -0.328 | 0.536 | 0.634 |
| CDT | -0.006 | -0.023 | 0.011 | 0.489 | -0.008 | -0.025 | 0.009 | 0.368 | -0.013 | -0.029 | 0.003 | 0.101 | **-0.017** | **-0.034** | **-0.001** | **0.041** |

***Note.*** Significant results (*P* < 0.05) are reported in bold.

**Table S6: Relationships between** **QSM values in limbic system subregions and multi-domain cognitive scores in all participants: multiple linear regression analysis**

| **Cognitive**  **Function** | **Left cingulate cortex** | | | | **Right cingulate cortex** | | | | **Left entorhinal cortex** | | | | **Right entorhinal cortex** | | | |
| --- | --- | --- | --- | --- | --- | --- | --- | --- | --- | --- | --- | --- | --- | --- | --- | --- |
|  | β | 95% CI | | *P* | β | 95% CI | | *P* | β | 95% CI | | *P* | β | 95% CI | | *P* |
| MoCA | -0.018 | -0.045 | 0.010 | 0.201 | -0.012 | -0.041 | 0.016 | 0.404 | -0.001 | -0.011 | 0.008 | 0.778 | -0.002 | -0.012 | 0.007 | 0.662 |
| MMSE | -0.006 | -0.030 | 0.017 | 0.597 | -0.007 | -0.032 | 0.018 | 0.565 | **-0.008** | **-0.016** | **0.000** | **0.049** | -0.005 | -0.013 | 0.003 | 0.241 |
| AVLT-immediate | -0.015 | -0.066 | 0.035 | 0.554 | -0.004 | -0.057 | 0.048 | 0.868 | -0.008 | -0.026 | 0.009 | 0.362 | -0.001 | -0.019 | 0.016 | 0.902 |
| AVLT-5min | -0.014 | -0.037 | 0.009 | 0.229 | -0.014 | -0.038 | 0.010 | 0.264 | -0.002 | -0.010 | 0.006 | 0.549 | -0.003 | -0.011 | 0.005 | 0.446 |
| AVLT-delay | -0.009 | -0.035 | 0.017 | 0.497 | -0.006 | -0.034 | 0.021 | 0.656 | -0.001 | -0.011 | 0.008 | 0.757 | -0.005 | -0.014 | 0.004 | 0.273 |
| AVLT-recall | -0.014 | -0.029 | 0.001 | 0.071 | -0.014 | -0.029 | 0.002 | 0.088 | -0.001 | -0.007 | 0.004 | 0.582 | -0.001 | -0.006 | 0.004 | 0.754 |
| TMT-A | **0.249** | **0.038** | **0.460** | **0.021** | **0.254** | **0.034** | **0.474** | **0.024** | 0.070 | -0.003 | 0.143 | 0.061 | **0.081** | **0.007** | **0.154** | **0.031** |
| DST-forward | 0.016 | -0.006 | 0.039 | 0.155 | 0.017 | -0.007 | 0.041 | 0.156 | -0.002 | -0.010 | 0.005 | 0.543 | 0.000 | -0.007 | 0.008 | 0.928 |
| DST-backward | -0.004 | -0.018 | 0.011 | 0.601 | -0.003 | -0.019 | 0.012 | 0.661 | -0.001 | -0.006 | 0.004 | 0.640 | -0.003 | -0.008 | 0.002 | 0.314 |
| GPT-R | 0.210 | -0.055 | 0.475 | 0.120 | 0.222 | -0.054 | 0.498 | 0.115 | **0.105** | **0.014** | **0.196** | **0.024** | 0.064 | -0.028 | 0.156 | 0.170 |
| GPT-L | 0.209 | -0.057 | 0.474 | 0.123 | 0.155 | -0.123 | 0.433 | 0.273 | 0.062 | -0.030 | 0.154 | 0.188 | 0.053 | -0.039 | 0.146 | 0.259 |
| CDT | **-0.010** | **-0.019** | **-0.001** | **0.024** | **-0.010** | **-0.019** | **-0.001** | **0.034** | -0.001 | -0.004 | 0.002 | 0.384 | -0.002 | -0.005 | 0.001 | 0.224 |
| **Cognitive**  **Function** | **Left parahippocampal** | | | | **Right parahippocampal** | | | |  |  |  |  |  |  |  |  |
|  | β | 95% CI | | *P* | β | 95% CI | | *P* |  |  |  |  |  |  |  |  |
| MoCA | -0.015 | -0.038 | 0.009 | 0.228 | -0.024 | -0.051 | 0.003 | 0.080 |  |  |  |  |  |  |  |  |
| MMSE | -0.013 | -0.034 | 0.007 | 0.197 | -0.022 | -0.045 | 0.001 | 0.063 |  |  |  |  |  |  |  |  |
| AVLT-immediate | -0.015 | -0.058 | 0.029 | 0.510 | -0.007 | -0.056 | 0.043 | 0.795 |  |  |  |  |  |  |  |  |
| AVLT-5min | -0.012 | -0.032 | 0.008 | 0.222 | -0.014 | -0.036 | 0.009 | 0.234 |  |  |  |  |  |  |  |  |
| AVLT-delay | -0.009 | -0.031 | 0.014 | 0.458 | -0.010 | -0.036 | 0.015 | 0.436 |  |  |  |  |  |  |  |  |
| AVLT-recall | -0.007 | -0.020 | 0.006 | 0.297 | -0.005 | -0.020 | 0.010 | 0.485 |  |  |  |  |  |  |  |  |
| TMT-A | **0.201** | **0.019** | **0.384** | **0.031** | **0.256** | **0.048** | **0.463** | **0.016** |  |  |  |  |  |  |  |  |
| DST-forward | 0.016 | -0.004 | 0.035 | 0.113 | 0.021 | -0.001 | 0.043 | 0.061 |  |  |  |  |  |  |  |  |
| DST-backward | 0.007 | -0.005 | 0.020 | 0.247 | -0.004 | -0.018 | 0.011 | 0.607 |  |  |  |  |  |  |  |  |
| GPT-R | 0.070 | -0.160 | 0.299 | 0.551 | 0.159 | -0.102 | 0.421 | 0.231 |  |  |  |  |  |  |  |  |
| GPT-L | 0.021 | -0.209 | 0.251 | 0.857 | 0.152 | -0.110 | 0.415 | 0.253 |  |  |  |  |  |  |  |  |
| CDT | 0.001 | -0.006 | 0.009 | 0.725 | **-0.009** | **-0.017** | **0.000** | **0.040** |  |  |  |  |  |  |  |  |

**Table S7: Relationships between QSM values in limbic system subregions and multi-domain cognitive scores in T2DM group: multiple linear regression analysis**

| Cognitive | **Left cingulate cortex** | | | | **Right cingulate cortex** | | | | **Left entorhinal cortex** | | | | **Right entorhinal cortex** | | | |
| --- | --- | --- | --- | --- | --- | --- | --- | --- | --- | --- | --- | --- | --- | --- | --- | --- |
| Function | β | 95% CI | | *P* | β | 95% CI | | *P* | β | 95% CI | | *P* | β | 95% CI | | *P* |
| MoCA | -0.017 | -0.064 | 0.030 | 0.477 | -0.012 | -0.059 | 0.035 | 0.618 | -0.001 | -0.016 | 0.014 | 0.887 | -0.008 | -0.024 | 0.009 | 0.370 |
| MMSE | -0.006 | -0.038 | 0.026 | 0.711 | -0.004 | -0.037 | 0.028 | 0.788 | -0.006 | -0.016 | 0.004 | 0.258 | **-0.013** | **-0.024** | **-0.001** | **0.029** |
| AVLT-immediate | -0.011 | -0.098 | 0.076 | 0.802 | -0.009 | -0.096 | 0.078 | 0.837 | -0.008 | -0.034 | 0.019 | 0.572 | -0.005 | -0.036 | 0.026 | 0.765 |
| AVLT-5min | -0.013 | -0.054 | 0.029 | 0.546 | -0.015 | -0.057 | 0.027 | 0.483 | -0.005 | -0.018 | 0.008 | 0.415 | -0.009 | -0.024 | 0.006 | 0.234 |
| AVLT-delay | -0.011 | -0.058 | 0.037 | 0.663 | -0.005 | -0.053 | 0.043 | 0.845 | 0.005 | -0.010 | 0.020 | 0.498 | -0.003 | -0.021 | 0.014 | 0.699 |
| AVLT-recall | -0.025 | -0.055 | 0.005 | 0.104 | -0.024 | -0.054 | 0.006 | 0.118 | -0.004 | -0.013 | 0.005 | 0.381 | -0.003 | -0.014 | 0.008 | 0.632 |
| TMT-A | 0.213 | -0.103 | 0.529 | 0.184 | 0.301 | -0.014 | 0.617 | 0.061 | 0.053 | -0.045 | 0.152 | 0.286 | 0.098 | -0.016 | 0.211 | 0.091 |
| DST-forward | 0.014 | -0.038 | 0.066 | 0.598 | 0.012 | -0.040 | 0.065 | 0.643 | -0.004 | -0.020 | 0.012 | 0.622 | 0.004 | -0.015 | 0.022 | 0.673 |
| DST-backward | -0.008 | -0.034 | 0.018 | 0.533 | -0.011 | -0.037 | 0.015 | 0.410 | 0.000 | -0.008 | 0.008 | 0.991 | -0.003 | -0.013 | 0.006 | 0.452 |
| GPT-R | -0.104 | -0.601 | 0.392 | 0.678 | 0.037 | -0.463 | 0.537 | 0.885 | 0.139 | -0.013 | 0.291 | 0.073 | 0.133 | -0.046 | 0.311 | 0.144 |
| GPT-L | -0.088 | -0.525 | 0.349 | 0.689 | -0.014 | -0.454 | 0.427 | 0.951 | 0.092 | -0.044 | 0.227 | 0.182 | 0.074 | -0.085 | 0.233 | 0.358 |
| CDT | **-0.018** | **-0.036** | **-0.001** | **0.034** | **-0.019** | **-0.036** | **-0.002** | **0.030** | -0.001 | -0.007 | 0.004 | 0.600 | -0.003 | -0.009 | 0.004 | 0.395 |
| Cognitive | **Left parahippocampal** | | | | **Right parahippocampal** | | | |  |  |  |  |  |  |  |  |
| Function | β | 95% CI | | *P* | β | 95% CI | | *P* |  |  |  |  |  |  |  |  |
| MoCA | 0.001 | -0.036 | 0.039 | 0.949 | -0.025 | -0.066 | 0.017 | 0.241 |  |  |  |  |  |  |  |  |
| MMSE | 0.005 | -0.021 | 0.030 | 0.717 | -0.024 | -0.052 | 0.004 | 0.092 |  |  |  |  |  |  |  |  |
| AVLT-immediate | -0.035 | -0.103 | 0.034 | 0.318 | -0.033 | -0.109 | 0.043 | 0.388 |  |  |  |  |  |  |  |  |
| AVLT-5min | -0.006 | -0.040 | 0.027 | 0.708 | -0.019 | -0.055 | 0.018 | 0.309 |  |  |  |  |  |  |  |  |
| AVLT-delay | -0.006 | -0.044 | 0.032 | 0.766 | -0.018 | -0.060 | 0.024 | 0.405 |  |  |  |  |  |  |  |  |
| AVLT-recall | -0.023 | -0.048 | 0.002 | 0.065 | -0.020 | -0.046 | 0.007 | 0.151 |  |  |  |  |  |  |  |  |
| TMT-A | 0.134 | -0.120 | 0.389 | 0.298 | 0.217 | -0.066 | 0.500 | 0.131 |  |  |  |  |  |  |  |  |
| DST-forward | 0.016 | -0.025 | 0.057 | 0.444 | 0.018 | -0.027 | 0.064 | 0.423 |  |  |  |  |  |  |  |  |
| DST-backward | -0.008 | -0.028 | 0.013 | 0.459 | -0.010 | -0.033 | 0.013 | 0.382 |  |  |  |  |  |  |  |  |
| GPT-R | -0.141 | -0.539 | 0.257 | 0.483 | 0.053 | -0.393 | 0.500 | 0.813 |  |  |  |  |  |  |  |  |
| GPT-L | 0.020 | -0.331 | 0.370 | 0.912 | 0.248 | -0.142 | 0.638 | 0.211 |  |  |  |  |  |  |  |  |
| CDT | 0.004 | -0.010 | 0.018 | 0.579 | -0.009 | -0.024 | 0.006 | 0.249 |  |  |  |  |  |  |  |  |

**Table S8. Moderating effect of HbA1c on the association between cortical regions QSM values and cognitive**

|  | | **Main Effects** | | **Interaction** | **VIF*_max_*** |
| --- | --- | --- | --- | --- | --- |
|  |  | QSM Values | HbA1c | QSM Values ✖ HbA1c |  |
| **MMSE** | | | | | |
| Right limbic cortex | *β* | -0.024 | -0.044 | -0.018 | 3.039 |
|  | 95% CI | (-0.059, 0.011) | (-0.201, 0.114) | (-0.031, -0.007) |  |
|  | *P* | 0.184 | 0.586 | **0.029** |  |
| **CDT** | | | | | |
| Left limbic cortex | *β* | -0.011 | -0.011 | -0.007 | 3.372 |
|  | 95% CI | (-0.023, 0.002) | (-0.068, 0.046) | (-0.013, -0.001) |  |
|  | *P* | 0.092 | 0.701 | **0.009** |  |
| Right limbic cortex | *β* | -0.014 | -0.008 | -0.010 | 3.000 |
|  | 95% CI | (-0.027, -0.002) | (-0.064, 0.048) | (-0.015, -0.004) |  |
|  | *P* | 0.024 | 0.776 | **<0.001** |  |

***Note.*** Only significant interactions (*P* < 0.05 with bootstrap 95% CI excluding zero) are shown.

**Table S9. Moderating effect of HbA1c on the association between limbic subregional QSM values and cognitive**

|  | | **Main Effects** | | **Interaction** | **VIF*_max_*** |
| --- | --- | --- | --- | --- | --- |
|  |  | QSM Values | HbA1c | QSM Values ✖ HbA1c |  |
| **MoCA** | | | | | |
| Right parahippocampal | *β* | -0.06 | -0.029 | -0.032 | 3.678 |
|  | 95% CI | (-0.106, -0.015) | (-0.221, 0.163) | (-0.053, -0.011) |  |
|  | *P* | 0.01 | 0.765 | **0.003** |  |
| **CDT** | | | | | |
| Right cingulate | *β* | -0.015 | 0.012 | -0.010 | 2.959 |
|  | 95% CI | (-0.028, -0.001) | (-0.046, 0.069) | (-0.017, -0.004) |  |
|  | *P* | 0.031 | 0.689 | **0.002** |  |
| Right entorhinal | *β* | -0.004 | -0.032 | -0.003 | 3.112 |
|  | 95% CI | (-0.008, 0.001) | (-0.090, 0.027) | (-0.004, -0.001) |  |
|  | *P* | 0.086 | 0.286 | **0.004** |  |

***Note.*** *Only significant interactions (P < 0.05 with bootstrap 95% CI excluding zero) are shown.*

| **Table S10. SVR prediction performance for TMT-A from limbic subregional QSM values in the T2DM group** | | | | | | | | | | |
| --- | --- | --- | --- | --- | --- | --- | --- | --- | --- | --- |
| **ROI** | **R²** | **R² 95% CI** | **RMSE** | **RMSE 95% CI** | **MAE** | **MAE 95% CI** | **Spearman’s *r*** | ***r* 95% CI** | ***P_FDR_*** |  |
| Left cingulate | 0.331 | [0.165, 0.488] | 17.204 | [13.057, 22.335] | 11.994 | [10.082, 14.414] | 0.634 | [0.509, 0.729] | <0.001 |  |
| Right cingulate | 0.332 | [0.153, 0.496] | 17.193 | [13.343, 22.221] | 12.316 | [10.409, 14.387] | 0.626 | [0.511, 0.723] | <0.001 |  |
| Left parahippocampal | 0.334 | [0.149, 0.517] | 17.159 | [12.926, 22.324] | 12.244 | [10.366, 14.638] | 0.631 | [0.523, 0.727] | <0.001 |  |
| Right parahippocampal | 0.305 | [0.153, 0.469] | 17.531 | [13.291, 22.405] | 12.174 | [10.135, 14.269] | 0.612 | [0.487, 0.712] | <0.001 |  |
| Left entorhinal | 0.222 | [0.018, 0.423] | 18.544 | [13.980, 24.049] | 12.841 | [10.650, 15.224] | 0.602 | [0.483, 0.696] | <0.001 |  |
| Right entorhinal | 0.323 | [0.158, 0.514] | 17.303 | [13.086, 22.310] | 12.233 | [10.374, 14.400] | 0.631 | [0.518, 0.721] | <0.001 |  |
| ***Note.*** SVR, support vector regression (RBF kernel); nested 5-fold cross-validation with inner-fold grid search. 95% CIs were estimated via 1,000-iteration bootstrap resampling of out-of-sample predictions. | | | | | | | | | | |

**Table S11 Demographic, clinical and cognitive characteristics of study participants in PSM cohort**

| **Variables** | **HC (n = 113)** | **T2DM (n = 113)** | **Statistic** | ***P*** | **SMD Before** | **SMD After** |
| --- | --- | --- | --- | --- | --- | --- |
| **Demographics** |  |  |  |  |  |  |
| Age (years) | 52.00 (43.00, 58.00) | 54.00 (44.00, 60.00) | Z = -1.09 | 0.275 | 0.259 | 0.139 |
| Sex (male/female) | 55/58 | 57/56 | χ² = 0.07 | 0.790 | -0.576 | -0.035 |
| Education (years) | 10.00 (9.00, 12.00) | 10.00 (8.00, 12.00) | Z = -0.45 | 0.650 | -0.019 | -0.09 |
| Diabetes duration (months) | - | 48.00 (12.00,120.00) |  |  |  |  |
| **Clinical data** |  |  |  |  |  |  |
| SBP (mmHg) | 127.50 (117.00, 138.00) | 127.00 (116.00, 140.00) | Z = -0.36 | 0.717 | 0.146 | -0.077 |
| DBP (mmHg) | 84.00 (75.00, 91.50) | 80.00 (72.00, 87.00) | Z = -2.15 | 0.032 | -0.125 | -0.334 |
| BMI (kg/m^2^) | 23.44 (21.88, 25.39) | 23.38 (21.33, 25.97) | Z = -0.08 | 0.933 | 0.139 | -0.029 |
| TC (mmol/L) | 4.97 (4.51, 5.64) | 4.86 (4.30, 5.65) | Z = -0.96 | 0.335 | -0.034 | -0.076 |
| TG (mmol/L) | 1.17 (0.91, 1.71) | 1.49 (1.04, 2.01) | Z = -3.24 | **0.001** | 0.134 | 0.143 |
| HDL-C (mmol/L) | 1.42 (1.17, 1.69) | 1.18 (0.97, 1.39) | Z = -4.93 | **< .001** | -0.883 | -0.736 |
| LDL-C (mmol/L) | 3.29 (2.73, 3.89) | 3.12 (2.57, 3.78) | Z=-1.21 | 0.227 | -0.095 | -0.143 |
| HbA1c (%) | 5.70 (5.40, 5.90) | 7.80 (6.70, 9.40) | Z = -10.87 | **< .001** | 1.275 | 1.221 |
| FPG (mmol/L) | 5.15 (4.89, 5.47) | 7.15 (5.87, 8.95) | Z = -8.46 | **< .001** | 1.675 | 1.646 |
| FINS (μIU/mL) | 8.36 (5.74, 12.05) | 6.58 (4.61, 11.00) | Z = -2.20 | **0.028** | -0.141 | -0.19 |
| **vascular risk factor** |  |  |  |  |  |  |
| Smoking, n (%) | 16 (14.16) | 16 (14.95) | χ² = 0.03 | 0.867 | 0.207 | 0.022 |
| Hypertension, n (%) | 6 (5.31) | 40 (35.40) | χ² = 31.55 | **< .001** | 0.805 | 0.802 |
| Alcohol, n (%) | 10 (8.85) | 16 (14.95) | χ² = 1.96 | 0.161 | 0.207 | 0.188 |
| **Cognitive scores** |  |  |  |  |  |  |
| MoCA | 26.00 (24.00, 27.00) | 25.00 (23.00, 27.00) | Z = -1.52 | 0.129 | - | - |
| MMSE | 28.00 (27.00, 29.00) | 28.00 (27.00, 29.00) | Z = -1.66 | 0.097 | - | - |
| AVLT-immediate | 21.00 (19.00, 24.00) | 20.00 (17.00, 24.00) | Z = -1.93 | 0.054 | - | - |
| AVLT-5min | 8.00 (6.50, 9.00) | 7.00 (6.00, 9.00) | Z = -2.31 | **0.021** | **-** | **-** |
| AVLT-delay | 8.00 (6.00, 9.50) | 7.00 (5.00, 9.00) | Z = -2.67 | **0.008** | **-** | **-** |
| AVLT-recall | 12.00 (11.00, 12.00) | 11.50 (10.00, 12.00) | Z = -1.74 | 0.081 | - | - |
| GPT-R (s) | 72.00 (63.00, 84.00) | 72.00 (65.00, 91.00) | Z = -1.39 | 0.164 | - | - |
| GPT-L (s) | 75.00 (67.00, 90.00) | 80.00 (70.00, 96.00) | Z = -1.86 | 0.062 | - | - |
| TMT-A (s) | 45.50 (33.00, 58.00) | 52.00 (42.25, 67.00) | Z = -3.65 | **< .001** | **-** | **-** |
| CDT | 4.00 (3.00, 4.00) | 3.00 (3.00, 4.00) | Z = -0.77 | 0.444 | - | - |
| DST forward | 7.00 (6.00, 8.00) | 7.00 (6.00, 8.00) | Z = -0.70 | 0.481 | - | - |
| DST backward | 4.00 (3.00, 5.00) | 4.00 (3.00, 5.00) | Z = -0.84 | 0.404 | - | - |

***Note****.* Data are expressed as median (Q1, Q3) and mean ± SD. The categorical variables are expressed as number (percentage).

***Abbreviations****.* BMI, body mass index; SBP, systolic blood pressure; DBP, diastolic blood pressure; HbA1c, hemoglobin A1c; FPG, fasting plasma glucose; FINS, fasting insulin; TG, triglycerides; TC, total cholesterol; LDL, low-density lipoprotein; MoCA, Montreal Cognitive Assessment; MMSE, Mini-Mental State Examination; AVLT, Auditory Verbal Learning Test; GPT, Grooved Pegboard Test; TMT-A: Trail Making Test-A; CDT, Clock Drawing Test; DST, Digit Span Test; SMD, standardized mean difference.

**Table S12 QSM values for cortex ROIs in both T2DM and HC groups in PSM cohort**

| **Region** | **T2DM** | **HC** | **statistic** | ***P* value** | ***P_FDR_*** |
| --- | --- | --- | --- | --- | --- |
| Left frontal cortex | -7.85 (-12.62, -1.74) | -7.72 (-14.29, -3.97) | -1.546 | 0.124 | 0.371 |
| Right frontal cortex | -6.77 (-10.46, -1.93) | -7.41 (-13.17, -2.15) | 1.551 | 0.122 | 0.371 |
| Left parietal cortex | -0.93 (-6.82, 2.79) | -2.91 (-8.11, 2.16) | 1.161 | 0.247 | 0.495 |
| Right parietal cortex | -2.00 (-7.16, 2.38) | -1.58 (-6.74, 2.53) | -0.424 | 0.672 | 0.806 |
| Left temporal cortex | -9.29 (-13.06, -3.28) | -9.86 (-15.53, -3.32) | 1.159 | 0.248 | 0.495 |
| Right temporal cortex | -10.76 (-15.85, -5.98) | -11.96 (-18.04, -5.49) | 0.570 | 0.569 | 0.759 |
| Left occipital cortex | -2.85 (-8.65, 2.77) | -3.68 (-9.94, 3.04) | 0.899 | 0.369 | 0.633 |
| Right occipital cortex | -1.90 (-6.81, 4.81) | -2.75 (-9.78, 5.45) | 0.757 | 0.450 | 0.674 |
| Left insular cortex | -14.23 (-21.12, -8.05) | -14.74 (-21.03, -7.58) | 0.208 | 0.835 | 0.835 |
| Right insular cortex | -12.40 (-18.62, -4.71) | -11.35 (-18.41, -6.36) | -0.213 | 0.832 | 0.835 |
| Left limbic cortex | -2.33 (-8.64, 4.12) | -4.73 (-12.86, 0.70) | 2.192 | **0.029** | 0.353 |
| Right limbic cortex | -2.73 (-8.48, 3.23) | -4.54 (-12.52, 1.56) | 1.826 | 0.069 | 0.371 |

***Note.*** Significant results (*P* < 0.05) are reported in bold. ^*^ Indicates *P_FDR_* < 0.05

**Table S13 QSM values for limbic subregional ROIs in both T2DM and HC groups in PSM cohort**

| **Region** | **T2DM** | **HC** | **statistic** | ***P* value** | ***P_FDR_*** |
| --- | --- | --- | --- | --- | --- |
| Left cingulate cortex | -2.60 (-9.54, 4.30) | -6.04 (-13.46, 2.68) | 2.795 | **0.006** | **0.019^*^** |
| Right cingulate cortex | -3.83 (-9.60, 3.22) | -5.77 (-12.96, 2.21) | 2.027 | **0.044** | 0.079 |
| Left entorhinal cortex | -15.16 (-35.52, 3.51) | -17.48 (-36.09, 1.09) | 0.052 | 0.958 | 0.958 |
| Right entorhinal cortex | -19.09 (-33.85, -0.07) | -16.18 (-35.64, 0.07) | -0.158 | 0.874 | 0.958 |
| Left parahippocampal | 10.15 (1.76, 17.38) | 8.44 (0.69, 14.41) | 1.945 | 0.053 | 0.079 |
| Right parahippocampal | 14.76 (8.56, 20.75) | 10.72 (5.71, 17.99) | 2.756 | **0.006** | **0.019^*^** |

***Note.*** Significant results (*P* < 0.05) are reported in bold. ^*^ Indicates *P_FDR_* < 0.05

**Table S14: Relationship between cortical QSM values and glycemic metabolic profiles in all participants within a PSM cohort**

|  | FPG | | | HbA1c | | | FINS | | |
| --- | --- | --- | --- | --- | --- | --- | --- | --- | --- |
|  | ***r*** | ***P*** | ***P_FDR_*** | ***r*** | ***P*** | ***P_FDR_*** | ***r*** | ***P*** | ***P_FDR_*** |
| Left frontal cortex | 0.125 | 0.070 | 0.211 | 0.083 | 0.232 | 0.821 | 0.114 | 0.104 | 0.250 |
| Right frontal cortex | 0.112 | 0.105 | 0.253 | 0.063 | 0.362 | 0.821 | 0.092 | 0.193 | 0.308 |
| Left parietal cortex | 0.071 | 0.307 | 0.461 | 0.057 | 0.410 | 0.821 | 0.089 | 0.207 | 0.308 |
| Right parietal cortex | 0.008 | 0.905 | 0.905 | 0.009 | 0.896 | 0.996 | 0.080 | 0.257 | 0.308 |
| Left temporal cortex | 0.048 | 0.490 | 0.535 | 0.000 | 0.996 | 0.996 | 0.083 | 0.240 | 0.308 |
| Right temporal cortex | 0.049 | 0.480 | 0.535 | 0.039 | 0.576 | 0.912 | 0.026 | 0.709 | 0.709 |
| Left occipital cortex | 0.072 | 0.299 | 0.461 | 0.034 | 0.621 | 0.912 | 0.095 | 0.176 | 0.308 |
| Right occipital cortex | **0.151** | **0.028** | 0.114 | 0.063 | 0.362 | 0.821 | 0.138 | 0.049 | 0.201 |
| Left insular cortex | 0.096 | 0.167 | 0.334 | 0.028 | 0.684 | 0.912 | **0.161** | **0.022** | 0.201 |
| Right insular cortex | 0.062 | 0.369 | 0.492 | 0.006 | 0.936 | 0.996 | 0.129 | 0.066 | 0.201 |
| Left limbic cortex | **0.189** | **0.006** | **0.036^*^** | 0.131 | 0.057 | 0.680 | 0.129 | 0.067 | 0.201 |
| Right limbic cortex | **0.200** | **0.004** | **0.036^*^** | 0.101 | 0.143 | 0.821 | 0.050 | 0.483 | 0.526 |

***Note.*** Significant results (*P* < 0.05) are reported in bold. * FDR correction, *P* <0.05

**Table S15: Relationship between limbic subregional QSM values and glycemic metabolic profiles in all participants within a PSM cohort**

|  | FPG |  |  | HbA1c |  |  | FINS |  |  |
| --- | --- | --- | --- | --- | --- | --- | --- | --- | --- |
|  | ***r*** | ***P*** | ***P_FDR_*** | ***r*** | ***P*** | ***P_FDR_*** | ***r*** | ***P*** | ***P_FDR_*** |
| Left cingulate cortex | **0.245** | **<.001** | **0.002^*^** | **0.171** | **0.013** | 0.077 | 0.123 | 0.081 | 0.485 |
| Right cingulate cortex | **0.223** | **0.001** | **0.003^*^** | 0.131 | 0.058 | 0.834 | 0.046 | 0.514 | 0.883 |
| Left entorhinal cortex | -0.028 | 0.686 | 0.686 | -0.026 | 0.706 | 0.348 | -0.010 | 0.883 | 0.547 |
| Right entorhinal cortex | 0.048 | 0.492 | 0.590 | -0.015 | 0.834 | 0.170 | -0.064 | 0.365 | 0.617 |
| Left parahippocampal | 0.082 | 0.238 | 0.358 | 0.083 | 0.232 | 0.834 | 0.091 | 0.196 | 0.547 |
| Right parahippocampal | 0.091 | 0.189 | 0.358 | 0.119 | 0.085 | 0.170 | 0.075 | 0.289 | 0.547 |

***Note.*** Significant results (*P* < 0.05) are reported in bold. * FDR correction, *P* <0.05

**Table S16: Relationships between QSM values in cortical regions and multi-domain cognitive scores in all participants: multiple linear regression analysis in a PSM cohort**

| Cognitive | **Left frontal cortex** | | | | **Right frontal cortex** | | | | **Left parietal cortex** | | | | **Right parietal cortex** | | | |
| --- | --- | --- | --- | --- | --- | --- | --- | --- | --- | --- | --- | --- | --- | --- | --- | --- |
| Function | β | 95% CI | | *P* | β | 95% CI | | *P* | β | 95% CI | | *P* | β | 95% CI | | *P* |
| MoCA | -0.032 | -0.072 | 0.007 | 0.107 | -0.037 | -0.076 | 0.003 | 0.067 | -0.025 | -0.065 | 0.016 | 0.233 | -0.035 | -0.078 | 0.008 | 0.110 |
| MMSE | 0.003 | -0.035 | 0.041 | 0.872 | -0.006 | -0.043 | 0.032 | 0.764 | -0.008 | -0.047 | 0.031 | 0.695 | -0.006 | -0.047 | 0.036 | 0.786 |
| AVLT-immediate | -0.015 | -0.089 | 0.059 | 0.688 | -0.005 | -0.078 | 0.069 | 0.902 | 0.011 | -0.064 | 0.087 | 0.767 | 0.004 | -0.076 | 0.085 | 0.917 |
| AVLT-5min | 0.003 | -0.030 | 0.036 | 0.854 | -0.001 | -0.034 | 0.032 | 0.954 | 0.008 | -0.026 | 0.042 | 0.652 | 0.011 | -0.025 | 0.047 | 0.560 |
| AVLT-delay | -0.013 | -0.051 | 0.026 | 0.523 | -0.019 | -0.058 | 0.019 | 0.327 | 0.000 | -0.040 | 0.040 | 0.996 | -0.005 | -0.048 | 0.037 | 0.816 |
| AVLT-recall | -0.020 | -0.045 | 0.004 | 0.103 | -0.016 | -0.040 | 0.008 | 0.191 | -0.006 | -0.031 | 0.019 | 0.644 | -0.015 | -0.041 | 0.012 | 0.273 |
| TMT-A | 0.178 | -0.157 | 0.513 | 0.296 | 0.216 | -0.117 | 0.550 | 0.203 | 0.209 | -0.134 | 0.552 | 0.231 | 0.296 | -0.068 | 0.661 | 0.110 |
| DST-forward | 0.024 | -0.013 | 0.061 | 0.206 | 0.023 | -0.014 | 0.060 | 0.223 | 0.025 | -0.013 | 0.063 | 0.189 | 0.029 | -0.011 | 0.069 | 0.152 |
| DST-backward | -0.001 | -0.022 | 0.019 | 0.905 | -0.003 | -0.024 | 0.017 | 0.738 | -0.005 | -0.026 | 0.016 | 0.612 | -0.010 | -0.033 | 0.012 | 0.363 |
| GPT-R | 0.021 | -0.372 | 0.413 | 0.918 | 0.039 | -0.354 | 0.432 | 0.845 | -0.081 | -0.484 | 0.322 | 0.691 | 0.009 | -0.421 | 0.439 | 0.968 |
| GPT-L | -0.154 | -0.533 | 0.224 | 0.422 | -0.090 | -0.469 | 0.288 | 0.638 | -0.037 | -0.424 | 0.350 | 0.852 | -0.024 | -0.437 | 0.389 | 0.908 |
| CDT | -0.010 | -0.023 | 0.004 | 0.151 | -0.012 | -0.025 | 0.001 | 0.080 | -0.011 | -0.025 | 0.002 | 0.101 | -0.011 | -0.026 | 0.003 | 0.125 |
| Cognitive | **Left temporal cortex** | | | | **Right temporal cortex** | | | | **Left occipital cortex** | | | | **Right occipital cortex** | | | |
| Function | β | 95% CI | | *P* | β | 95% CI | | *P* | β | 95% CI | | *P* | β | 95% CI | | *P* |
| MoCA | -0.020 | -0.058 | 0.018 | 0.304 | -0.014 | -0.052 | 0.024 | 0.476 | -0.020 | -0.056 | 0.015 | 0.258 | -0.018 | -0.051 | 0.016 | 0.300 |
| MMSE | -0.006 | -0.042 | 0.031 | 0.766 | 0.008 | -0.028 | 0.044 | 0.663 | 0.001 | -0.033 | 0.035 | 0.963 | -0.006 | -0.038 | 0.026 | 0.707 |
| AVLT-immediate | 0.020 | -0.052 | 0.091 | 0.589 | 0.028 | -0.043 | 0.099 | 0.434 | 0.022 | -0.043 | 0.088 | 0.503 | 0.027 | -0.035 | 0.090 | 0.390 |
| AVLT-5min | 0.018 | -0.014 | 0.050 | 0.280 | 0.023 | -0.009 | 0.055 | 0.154 | 0.020 | -0.009 | 0.050 | 0.181 | 0.012 | -0.016 | 0.040 | 0.397 |
| AVLT-delay | 0.010 | -0.028 | 0.048 | 0.599 | 0.012 | -0.026 | 0.049 | 0.532 | 0.009 | -0.026 | 0.044 | 0.611 | 0.005 | -0.028 | 0.038 | 0.772 |
| AVLT-recall | -0.014 | -0.038 | 0.009 | 0.227 | -0.010 | -0.033 | 0.013 | 0.386 | -0.010 | -0.031 | 0.012 | 0.377 | -0.007 | -0.027 | 0.014 | 0.514 |
| TMT-A | 0.015 | -0.312 | 0.342 | 0.930 | -0.099 | -0.421 | 0.224 | 0.547 | 0.121 | -0.178 | 0.420 | 0.426 | 0.076 | -0.206 | 0.358 | 0.594 |
| DST-forward | 0.018 | -0.018 | 0.054 | 0.331 | 0.014 | -0.022 | 0.049 | 0.455 | 0.021 | -0.012 | 0.054 | 0.212 | 0.017 | -0.014 | 0.049 | 0.275 |
| DST-backward | -0.011 | -0.031 | 0.009 | 0.267 | -0.009 | -0.029 | 0.011 | 0.364 | -0.008 | -0.026 | 0.010 | 0.380 | -0.008 | -0.025 | 0.009 | 0.354 |
| GPT-R | -0.084 | -0.467 | 0.299 | 0.665 | -0.150 | -0.528 | 0.228 | 0.434 | 0.035 | -0.316 | 0.385 | 0.845 | 0.023 | -0.307 | 0.353 | 0.891 |
| GPT-L | -0.162 | -0.530 | 0.207 | 0.389 | -0.205 | -0.569 | 0.159 | 0.268 | -0.091 | -0.427 | 0.245 | 0.594 | -0.068 | -0.384 | 0.248 | 0.672 |
| CDT | -0.007 | -0.020 | 0.006 | 0.305 | -0.008 | -0.021 | 0.005 | 0.219 | -0.009 | -0.021 | 0.003 | 0.153 | -0.007 | -0.018 | 0.004 | 0.214 |
| Cognitive | **Left insular cortex** | | | | **Right insular cortex** | | | | **Left limbic cortex** | | | | **Right limbic cortex** | | | |
| Function | β | 95% CI | | *P* | β | 95% CI | | *P* | β | 95% CI | | *P* | β | 95% CI | | *P* |
| MoCA | -0.007 | -0.042 | 0.028 | 0.690 | -0.019 | -0.057 | 0.018 | 0.303 | -0.018 | -0.050 | 0.014 | 0.273 | -0.010 | -0.043 | 0.023 | 0.549 |
| MMSE | 0.007 | -0.026 | 0.041 | 0.658 | 0.003 | -0.033 | 0.038 | 0.887 | -0.015 | -0.045 | 0.016 | 0.345 | -0.012 | -0.043 | 0.020 | 0.468 |
| AVLT-immediate | 0.036 | -0.029 | 0.101 | 0.282 | 0.043 | -0.026 | 0.113 | 0.219 | -0.006 | -0.066 | 0.054 | 0.849 | 0.011 | -0.051 | 0.073 | 0.725 |
| AVLT-5min | 0.022 | -0.007 | 0.051 | 0.141 | 0.022 | -0.009 | 0.053 | 0.169 | -0.003 | -0.030 | 0.024 | 0.816 | -0.006 | -0.034 | 0.022 | 0.661 |
| AVLT-delay | 0.013 | -0.021 | 0.048 | 0.446 | 0.008 | -0.029 | 0.044 | 0.679 | -0.003 | -0.035 | 0.028 | 0.833 | -0.010 | -0.042 | 0.023 | 0.549 |
| AVLT-recall | -0.008 | -0.030 | 0.013 | 0.439 | 0.000 | -0.022 | 0.023 | 0.984 | -0.014 | -0.033 | 0.005 | 0.155 | -0.008 | -0.028 | 0.011 | 0.400 |
| TMT-A | 0.038 | -0.256 | 0.332 | 0.799 | 0.044 | -0.268 | 0.357 | 0.780 | 0.176 | -0.093 | 0.445 | 0.198 | 0.173 | -0.103 | 0.449 | 0.219 |
| DST-forward | 0.032 | -0.001 | 0.064 | 0.056 | 0.021 | -0.013 | 0.056 | 0.230 | 0.012 | -0.017 | 0.042 | 0.410 | 0.018 | -0.012 | 0.049 | 0.234 |
| DST-backward | 0.003 | -0.015 | 0.021 | 0.763 | -0.004 | -0.023 | 0.016 | 0.714 | -0.004 | -0.021 | 0.012 | 0.619 | -0.009 | -0.025 | 0.008 | 0.322 |
| GPT-R | -0.070 | -0.416 | 0.275 | 0.688 | -0.175 | -0.543 | 0.193 | 0.350 | 0.089 | -0.227 | 0.405 | 0.580 | 0.070 | -0.254 | 0.395 | 0.670 |
| GPT-L | -0.133 | -0.466 | 0.200 | 0.431 | -0.203 | -0.556 | 0.151 | 0.259 | 0.005 | -0.299 | 0.309 | 0.973 | 0.024 | -0.289 | 0.336 | 0.882 |
| CDT | -0.010 | -0.022 | 0.002 | 0.106 | -0.011 | -0.023 | 0.002 | 0.089 | **-0.012** | **-0.022** | **-0.001** | **0.034** | **-0.014** | **-0.025** | **-0.003** | **0.011** |

***Note.*** Significant results (*P* < 0.05) are reported in bold.

**Table S17: Relationships between QSM values in cortical regions and multi-domain cognitive scores in T2DM group: multiple linear regression analysis in a PSM cohort**

| Cognitive | **Left frontal cortex** | | | | **Right frontal cortex** | | | | **Left parietal cortex** | | | | **Right parietal cortex** | | | |
| --- | --- | --- | --- | --- | --- | --- | --- | --- | --- | --- | --- | --- | --- | --- | --- | --- |
| Function | β | 95% CI | | *P* | β | 95% CI | | *P* | β | 95% CI | | *P* | β | 95% CI | | *P* |
| MoCA | -0.035 | -0.092 | 0.021 | 0.219 | -0.058 | -0.117 | 0.001 | 0.053 | -0.040 | -0.100 | 0.020 | 0.192 | -0.036 | -0.100 | 0.029 | 0.273 |
| MMSE | -0.002 | -0.043 | 0.039 | 0.928 | -0.007 | -0.049 | 0.036 | 0.763 | -0.003 | -0.047 | 0.040 | 0.876 | 0.000 | -0.046 | 0.047 | 0.994 |
| AVLT-immediate | -0.030 | -0.135 | 0.075 | 0.570 | -0.044 | -0.154 | 0.067 | 0.434 | 0.006 | -0.106 | 0.119 | 0.909 | -0.012 | -0.132 | 0.108 | 0.840 |
| AVLT-5min | -0.001 | -0.051 | 0.049 | 0.973 | -0.017 | -0.069 | 0.035 | 0.520 | 0.006 | -0.047 | 0.059 | 0.810 | 0.005 | -0.052 | 0.062 | 0.861 |
| AVLT-delay | -0.012 | -0.070 | 0.046 | 0.675 | -0.034 | -0.095 | 0.027 | 0.269 | -0.001 | -0.062 | 0.061 | 0.984 | -0.006 | -0.072 | 0.060 | 0.857 |
| AVLT-recall | **-0.042** | **-0.082** | **-0.002** | **0.038** | **-0.043** | **-0.084** | **-0.001** | **0.044** | -0.025 | -0.068 | 0.019 | 0.257 | -0.039 | -0.085 | 0.006 | 0.088 |
| TMT-A | 0.394 | -0.113 | 0.901 | 0.126 | **0.555** | **0.027** | **1.083** | **0.039** | 0.407 | -0.136 | 0.950 | 0.140 | 0.452 | -0.127 | 1.030 | 0.124 |
| DST-forward | 0.016 | -0.051 | 0.083 | 0.635 | 0.014 | -0.056 | 0.084 | 0.696 | 0.019 | -0.053 | 0.091 | 0.601 | 0.027 | -0.049 | 0.103 | 0.479 |
| DST-backward | -0.010 | -0.041 | 0.020 | 0.500 | -0.007 | -0.039 | 0.024 | 0.647 | -0.010 | -0.042 | 0.022 | 0.533 | -0.011 | -0.045 | 0.023 | 0.513 |
| GPT-R | 0.007 | -0.645 | 0.659 | 0.983 | 0.093 | -0.596 | 0.782 | 0.788 | -0.084 | -0.782 | 0.614 | 0.812 | -0.090 | -0.835 | 0.654 | 0.810 |
| GPT-L | -0.004 | -0.591 | 0.584 | 0.990 | 0.176 | -0.443 | 0.795 | 0.574 | 0.170 | -0.452 | 0.791 | 0.589 | 0.145 | -0.521 | 0.811 | 0.667 |
| CDT | -0.014 | -0.035 | 0.007 | 0.200 | -0.020 | -0.042 | 0.002 | 0.077 | -0.016 | -0.038 | 0.007 | 0.171 | -0.018 | -0.043 | 0.006 | 0.130 |
| Cognitive | **Left temporal cortex** | | | | **Right temporal cortex** | | | | **Left occipital cortex** | | | | **Right occipital cortex** | | | |
| Function | β | 95% CI | | *P* | β | 95% CI | | *P* | β | 95% CI | | *P* | β | 95% CI | | *P* |
| MoCA | -0.032 | -0.091 | 0.027 | 0.285 | -0.024 | -0.086 | 0.039 | 0.455 | -0.025 | -0.078 | 0.027 | 0.341 | -0.026 | -0.076 | 0.024 | 0.310 |
| MMSE | 0.013 | -0.029 | 0.056 | 0.535 | 0.024 | -0.021 | 0.069 | 0.286 | 0.006 | -0.032 | 0.044 | 0.747 | -0.003 | -0.039 | 0.033 | 0.870 |
| AVLT-immediate | -0.016 | -0.126 | 0.094 | 0.775 | 0.013 | -0.103 | 0.129 | 0.822 | -0.001 | -0.098 | 0.097 | 0.992 | -0.003 | -0.096 | 0.091 | 0.953 |
| AVLT-5min | 0.007 | -0.045 | 0.058 | 0.800 | 0.021 | -0.033 | 0.076 | 0.444 | 0.016 | -0.030 | 0.061 | 0.502 | 0.007 | -0.037 | 0.051 | 0.756 |
| AVLT-delay | -0.004 | -0.065 | 0.056 | 0.891 | 0.000 | -0.064 | 0.064 | 0.992 | 0.010 | -0.044 | 0.063 | 0.713 | 0.000 | -0.051 | 0.052 | 0.992 |
| AVLT-recall | -0.039 | -0.080 | 0.003 | 0.066 | **-0.046** | **-0.089** | **-0.003** | **0.038** | -0.019 | -0.056 | 0.019 | 0.323 | -0.024 | -0.059 | 0.012 | 0.192 |
| TMT-A | 0.200 | -0.338 | 0.738 | 0.463 | 0.109 | -0.456 | 0.674 | 0.702 | 0.286 | -0.180 | 0.751 | 0.227 | 0.162 | -0.287 | 0.611 | 0.476 |
| DST-forward | 0.018 | -0.052 | 0.089 | 0.606 | 0.022 | -0.051 | 0.096 | 0.549 | 0.016 | -0.046 | 0.077 | 0.616 | 0.006 | -0.054 | 0.065 | 0.852 |
| DST-backward | -0.014 | -0.045 | 0.018 | 0.383 | -0.008 | -0.041 | 0.026 | 0.650 | -0.015 | -0.043 | 0.013 | 0.282 | -0.015 | -0.041 | 0.012 | 0.266 |
| GPT-R | -0.118 | -0.802 | 0.567 | 0.734 | -0.223 | -0.940 | 0.493 | 0.538 | 0.060 | -0.536 | 0.655 | 0.843 | -0.082 | -0.652 | 0.488 | 0.776 |
| GPT-L | -0.044 | -0.658 | 0.571 | 0.888 | -0.100 | -0.752 | 0.553 | 0.762 | 0.013 | -0.518 | 0.543 | 0.962 | -0.057 | -0.565 | 0.450 | 0.823 |
| CDT | -0.011 | -0.034 | 0.011 | 0.312 | -0.015 | -0.039 | 0.008 | 0.192 | -0.016 | -0.035 | 0.004 | 0.114 | -0.017 | -0.036 | 0.001 | 0.070 |
| Cognitive | **Left insular cortex** | | | | **Right insular cortex** | | | | **Left limbic cortex** | | | | **Right limbic cortex** | | | |
| Function | β | 95% CI | | *P* | β | 95% CI | | *P* | β | 95% CI | | *P* | β | 95% CI | | *P* |
| MoCA | -0.006 | -0.057 | 0.045 | 0.822 | -0.030 | -0.081 | 0.022 | 0.261 | -0.033 | -0.081 | 0.014 | 0.168 | -0.038 | -0.089 | 0.013 | 0.147 |
| MMSE | 0.003 | -0.033 | 0.040 | 0.852 | 0.001 | -0.035 | 0.038 | 0.942 | -0.009 | -0.043 | 0.025 | 0.588 | -0.021 | -0.057 | 0.015 | 0.251 |
| AVLT-immediate | 0.038 | -0.056 | 0.132 | 0.424 | 0.040 | -0.056 | 0.136 | 0.410 | -0.015 | -0.104 | 0.074 | 0.737 | -0.019 | -0.115 | 0.076 | 0.689 |
| AVLT-5min | 0.029 | -0.016 | 0.073 | 0.202 | 0.016 | -0.029 | 0.062 | 0.475 | -0.011 | -0.053 | 0.031 | 0.609 | -0.028 | -0.073 | 0.016 | 0.209 |
| AVLT-delay | 0.029 | -0.022 | 0.081 | 0.265 | 0.012 | -0.041 | 0.065 | 0.664 | -0.001 | -0.050 | 0.048 | 0.962 | -0.025 | -0.077 | 0.027 | 0.339 |
| AVLT-recall | -0.022 | -0.057 | 0.013 | 0.210 | -0.012 | -0.047 | 0.024 | 0.508 | **-0.033** | **-0.064** | **-0.001** | **0.045** | -0.032 | -0.066 | 0.002 | 0.062 |
| TMT-A | 0.206 | -0.244 | 0.656 | 0.366 | 0.336 | -0.116 | 0.788 | 0.143 | 0.362 | -0.052 | 0.776 | 0.086 | 0.343 | -0.102 | 0.788 | 0.129 |
| DST-forward | 0.024 | -0.036 | 0.083 | 0.431 | 0.018 | -0.042 | 0.078 | 0.546 | 0.001 | -0.054 | 0.057 | 0.958 | 0.015 | -0.044 | 0.074 | 0.621 |
| DST-backward | 0.000 | -0.027 | 0.027 | 0.998 | -0.011 | -0.037 | 0.016 | 0.440 | -0.009 | -0.034 | 0.016 | 0.477 | -0.019 | -0.045 | 0.008 | 0.167 |
| GPT-R | -0.081 | -0.659 | 0.497 | 0.781 | -0.142 | -0.732 | 0.447 | 0.632 | 0.172 | -0.361 | 0.704 | 0.523 | 0.275 | -0.294 | 0.845 | 0.340 |
| GPT-L | 0.022 | -0.502 | 0.545 | 0.934 | -0.014 | -0.540 | 0.512 | 0.959 | 0.107 | -0.368 | 0.583 | 0.655 | 0.166 | -0.345 | 0.677 | 0.520 |
| CDT | -0.011 | -0.030 | 0.008 | 0.242 | -0.013 | -0.032 | 0.006 | 0.172 | **-0.018** | **-0.036** | **-0.001** | **0.037** | **-0.025** | **-0.043** | **-0.007** | **0.007** |

***Note.*** Significant results (*P* < 0.05) are reported in bold.

**Table S18: Relationships between QSM values in limbic system subregions and multi-domain cognitive scores in all participants: multiple linear regression analysis in a PSM cohort**

| Cognitive | **Left cingulate** | | | | **Right cingulate** | | | | **Left entorhinal** | | | | **Right entorhinal** | | | |
| --- | --- | --- | --- | --- | --- | --- | --- | --- | --- | --- | --- | --- | --- | --- | --- | --- |
| Function | β | 95% CI | | *P* | β | 95% CI | | *P* | β | 95% CI | | *P* | β | 95% CI | | *P* |
| MoCA | -0.028 | -0.061 | 0.005 | 0.095 | -0.015 | -0.049 | 0.019 | 0.392 | 0.003 | -0.009 | 0.014 | 0.659 | 0.003 | -0.009 | 0.015 | 0.616 |
| MMSE | -0.008 | -0.039 | 0.024 | 0.635 | -0.003 | -0.036 | 0.029 | 0.840 | -0.006 | -0.017 | 0.004 | 0.243 | -0.004 | -0.015 | 0.007 | 0.452 |
| AVLT-immediate | -0.011 | -0.073 | 0.052 | 0.731 | 0.009 | -0.056 | 0.074 | 0.780 | 0.000 | -0.021 | 0.021 | 0.985 | 0.006 | -0.016 | 0.027 | 0.614 |
| AVLT-5min | -0.008 | -0.036 | 0.020 | 0.581 | -0.007 | -0.036 | 0.022 | 0.648 | 0.000 | -0.009 | 0.010 | 0.938 | -0.001 | -0.011 | 0.009 | 0.869 |
| AVLT-delay | -0.012 | -0.044 | 0.021 | 0.488 | -0.009 | -0.042 | 0.025 | 0.621 | 0.002 | -0.009 | 0.013 | 0.726 | -0.003 | -0.015 | 0.008 | 0.583 |
| AVLT-recall | -0.016 | -0.035 | 0.004 | 0.126 | -0.014 | -0.034 | 0.007 | 0.189 | -0.002 | -0.009 | 0.005 | 0.615 | 0.001 | -0.006 | 0.009 | 0.676 |
| TMT-A | 0.110 | -0.169 | 0.388 | 0.439 | 0.133 | -0.156 | 0.421 | 0.365 | 0.053 | -0.041 | 0.148 | 0.269 | 0.041 | -0.058 | 0.139 | 0.417 |
| DST-forward | 0.023 | -0.008 | 0.053 | 0.147 | 0.020 | -0.012 | 0.052 | 0.210 | -0.003 | -0.014 | 0.007 | 0.517 | 0.001 | -0.010 | 0.012 | 0.877 |
| DST-backward | -0.005 | -0.022 | 0.012 | 0.547 | -0.007 | -0.025 | 0.011 | 0.426 | -0.001 | -0.007 | 0.005 | 0.752 | -0.004 | -0.010 | 0.002 | 0.202 |
| GPT-R | -0.020 | -0.347 | 0.307 | 0.905 | 0.039 | -0.299 | 0.378 | 0.820 | 0.080 | -0.030 | 0.191 | 0.154 | 0.031 | -0.085 | 0.146 | 0.601 |
| GPT-L | -0.048 | -0.362 | 0.266 | 0.763 | -0.020 | -0.345 | 0.305 | 0.903 | 0.031 | -0.077 | 0.138 | 0.573 | 0.015 | -0.096 | 0.126 | 0.789 |
| CDT | **-0.014** | **-0.025** | **-0.003** | **0.017** | **-0.015** | **-0.026** | **-0.003** | **0.011** | -0.002 | -0.006 | 0.002 | 0.361 | -0.003 | -0.006 | 0.001 | 0.205 |
| Cognitive | **Left parahippocampal** | | | | **Right parahippocampal** | | | |  |  |  |  |  |  |  |  |
| Function | β | 95% CI | | *P* | β | 95% CI | | *P* |  |  |  |  |  |  |  |  |
| MoCA | -0.014 | -0.043 | 0.015 | 0.338 | -0.028 | -0.059 | 0.002 | 0.066 |  |  |  |  |  |  |  |  |
| MMSE | -0.016 | -0.043 | 0.011 | 0.250 | -0.024 | -0.052 | 0.005 | 0.110 |  |  |  |  |  |  |  |  |
| AVLT-immediate | -0.013 | -0.066 | 0.040 | 0.632 | -0.019 | -0.076 | 0.038 | 0.518 |  |  |  |  |  |  |  |  |
| AVLT-5min | -0.001 | -0.025 | 0.023 | 0.940 | -0.014 | -0.039 | 0.012 | 0.296 |  |  |  |  |  |  |  |  |
| AVLT-delay | -0.005 | -0.033 | 0.023 | 0.733 | -0.016 | -0.046 | 0.014 | 0.305 |  |  |  |  |  |  |  |  |
| AVLT-recall | -0.010 | -0.028 | 0.008 | 0.284 | -0.007 | -0.025 | 0.011 | 0.459 |  |  |  |  |  |  |  |  |
| TMT-A | 0.189 | -0.051 | 0.429 | 0.122 | 0.223 | -0.034 | 0.481 | 0.088 |  |  |  |  |  |  |  |  |
| DST-forward | 0.015 | -0.012 | 0.041 | 0.278 | 0.025 | -0.004 | 0.053 | 0.087 |  |  |  |  |  |  |  |  |
| DST-backward | 0.005 | -0.010 | 0.019 | 0.544 | 0.002 | -0.013 | 0.018 | 0.762 |  |  |  |  |  |  |  |  |
| GPT-R | 0.021 | -0.261 | 0.304 | 0.881 | 0.054 | -0.249 | 0.357 | 0.727 |  |  |  |  |  |  |  |  |
| GPT-L | 0.023 | -0.248 | 0.294 | 0.867 | 0.175 | -0.115 | 0.464 | 0.235 |  |  |  |  |  |  |  |  |
| CDT | 0.002 | -0.008 | 0.012 | 0.694 | -0.007 | -0.018 | 0.003 | 0.165 |  |  |  |  |  |  |  |  |

**Table S19: Relationships between QSM values in limbic system subregions and multi-domain cognitive scores in T2DM group multiple linear regression analysis in a PSM cohort**

| Cognitive  Function | **Left cingulate** | | | | **Right cingulate** | | | | **Left entorhinal** | | | | **Right entorhinal** | | | |
| --- | --- | --- | --- | --- | --- | --- | --- | --- | --- | --- | --- | --- | --- | --- | --- | --- |
|  | *β* | 95% CI | | *P* | *β* | 95% CI | | *P* | *β* | 95% CI | | *P* | *β* | 95% CI | | *P* |
| MoCA | -0.046 | -0.099 | 0.007 | 0.090 | -0.032 | -0.086 | 0.021 | 0.237 | -0.004 | -0.020 | 0.012 | 0.611 | -0.008 | -0.026 | 0.010 | 0.393 |
| MMSE | -0.003 | -0.040 | 0.035 | 0.893 | 0.002 | -0.036 | 0.040 | 0.902 | -0.005 | -0.016 | 0.006 | 0.375 | **-0.014** | **-0.026** | **-0.001** | **0.033** |
| AVLT-immediate | -0.006 | -0.106 | 0.094 | 0.904 | -0.001 | -0.102 | 0.099 | 0.981 | -0.008 | -0.038 | 0.021 | 0.566 | -0.011 | -0.045 | 0.023 | 0.519 |
| AVLT-5min | -0.012 | -0.058 | 0.035 | 0.623 | -0.018 | -0.065 | 0.028 | 0.440 | -0.006 | -0.020 | 0.007 | 0.354 | -0.012 | -0.028 | 0.003 | 0.125 |
| AVLT-delay | -0.014 | -0.068 | 0.041 | 0.617 | -0.017 | -0.071 | 0.038 | 0.545 | 0.003 | -0.013 | 0.019 | 0.715 | -0.009 | -0.028 | 0.009 | 0.323 |
| AVLT-recall | -0.034 | -0.070 | 0.002 | 0.060 | -0.034 | -0.069 | 0.002 | 0.061 | -0.006 | -0.017 | 0.004 | 0.237 | -0.005 | -0.017 | 0.008 | 0.448 |
| TMT-A | 0.254 | -0.213 | 0.721 | 0.283 | 0.300 | -0.166 | 0.767 | 0.205 | 0.117 | -0.020 | 0.253 | 0.094 | 0.085 | -0.075 | 0.245 | 0.296 |
| DST-forward | 0.016 | -0.046 | 0.078 | 0.616 | 0.013 | -0.049 | 0.075 | 0.681 | -0.005 | -0.023 | 0.013 | 0.577 | 0.003 | -0.018 | 0.024 | 0.766 |
| DST-backward | -0.011 | -0.039 | 0.017 | 0.437 | -0.017 | -0.045 | 0.010 | 0.218 | -0.001 | -0.009 | 0.008 | 0.875 | -0.005 | -0.015 | 0.004 | 0.274 |
| GPT-R | -0.073 | -0.668 | 0.523 | 0.809 | 0.101 | -0.496 | 0.699 | 0.737 | 0.165 | -0.008 | 0.337 | 0.061 | 0.147 | -0.056 | 0.349 | 0.154 |
| GPT-L | -0.092 | -0.621 | 0.437 | 0.729 | 0.022 | -0.511 | 0.554 | 0.936 | 0.113 | -0.043 | 0.269 | 0.154 | 0.087 | -0.096 | 0.270 | 0.346 |
| CDT | **-0.024** | **-0.043** | **-0.005** | **0.015** | -0.027 | -0.046 | -0.008 | 0.006 | -0.003 | -0.009 | 0.003 | 0.307 | -0.005 | -0.011 | 0.002 | 0.167 |
| Cognitive  Function | **Left parahippocampal** | | | | **Right parahippocampal** | | | |  |  |  |  |  |  |  |  |
|  | *β* | 95% CI | | *P* | *β* | 95% CI | | *P* |  |  |  |  |  |  |  |  |
| MoCA | -0.006 | -0.050 | 0.037 | 0.772 | -0.034 | -0.080 | 0.012 | 0.140 |  |  |  |  |  |  |  |  |
| MMSE | 0.002 | -0.028 | 0.033 | 0.878 | -0.030 | -0.062 | 0.003 | 0.072 |  |  |  |  |  |  |  |  |
| AVLT-immediate | -0.025 | -0.105 | 0.054 | 0.527 | -0.020 | -0.106 | 0.066 | 0.645 |  |  |  |  |  |  |  |  |
| AVLT-5min | 0.013 | -0.024 | 0.051 | 0.492 | -0.011 | -0.052 | 0.029 | 0.574 |  |  |  |  |  |  |  |  |
| AVLT-delay | -0.003 | -0.047 | 0.041 | 0.887 | -0.023 | -0.070 | 0.024 | 0.336 |  |  |  |  |  |  |  |  |
| AVLT-recall | -0.029 | -0.059 | 0.001 | 0.059 | -0.018 | -0.049 | 0.013 | 0.252 |  |  |  |  |  |  |  |  |
| TMT-A | 0.308 | -0.069 | 0.685 | 0.108 | 0.229 | -0.186 | 0.645 | 0.276 |  |  |  |  |  |  |  |  |
| DST-forward | 0.016 | -0.034 | 0.067 | 0.522 | 0.022 | -0.032 | 0.076 | 0.425 |  |  |  |  |  |  |  |  |
| DST-backward | -0.003 | -0.026 | 0.020 | 0.789 | -0.005 | -0.030 | 0.019 | 0.661 |  |  |  |  |  |  |  |  |
| GPT-R | -0.063 | -0.548 | 0.421 | 0.796 | 0.125 | -0.404 | 0.655 | 0.640 |  |  |  |  |  |  |  |  |
| GPT-L | 0.103 | -0.327 | 0.534 | 0.635 | 0.337 | -0.129 | 0.803 | 0.154 |  |  |  |  |  |  |  |  |
| CDT | 0.002 | -0.014 | 0.018 | 0.794 | -0.011 | -0.028 | 0.006 | 0.211 |  |  |  |  |  |  |  |  |

**Table S20: Moderating effect of HbA1c on the association between cortical and limbic subregion**

**QSM values and cognitive scores in PSM matched cohort**

|  | | **Main Effects** | | **Interaction** | **VIF*_max_*** |
| --- | --- | --- | --- | --- | --- |
|  |  | QSM Values | HbA1c | QSM Values ✖ HbA1c |  |
| **CDT** | | | | | |
| Right limbic cortex | *β* | -0.015 | -0.021 | -0.010 | 3.289 |
|  | 95% CI | (-0.032, 0.002) | (-0.086, 0.044) | (-0.016, -0.003) |  |
|  | *P* | 0.092 | 0.525 | **0.004** |  |
| **MoCA** | | | | | |
| Right parahippocampal | *β* | -0.069 | -0.121 | -0.032 | 3.842 |
|  | 95% CI | (-0.124, -0.014) | (-0.327,0.085) | (-0.054, -0.010) |  |
|  | *P* | 0.014 | 0.247 | **0.005** |  |
| **CDT** |  |  |  |  |  |
| Right cingulate | *β* | -0.019 | 0.002 | -0.011 | 3.128 |
|  | 95% CI | (-0.037, -0.001) | (-0.064, 0.067) | (-0.019, -0.004) |  |
|  | *P* | 0.036 | 0.954 | **0.003** |  |

***Note.*** Significant results (*P* < 0.05) are reported in bold.

**Table S21:** **Associations between cortical and limbic subregion QSM values and TMT-A scores**

**at different HbA1c levels in PSM cohort**

|  | **Moderator Level**  **(HbA1c)** | **Slope** | **95% CI** | | ***P* value** |
| --- | --- | --- | --- | --- | --- |
| **CDT** | | | | | |
| Right limbic cortex | Low (-1 SD) | 0.005 | -0.011 | 0.022 | 0.508 |
|  | Mean | -0.015 | -0.032 | 0.002 | 0.092 |
|  | High (+1 SD) | -0.035 | -0.061 | -0.009 | **0.009** |
| **MoCA** | | | | | |
| Right parahippocampal | Low (-1 SD) | -0.001 | -0.049 | 0.047 | 0.967 |
|  | Mean | -0.069 | -0.124 | -0.014 | **0.014** |
|  | High (+1 SD) | -0.137 | -0.227 | -0.047 | **0.003** |
| **CDT** | | | | | |
| Right cingulate | Low (-1 SD) | 0.004 | -0.012 | 0.021 | 0.605 |
|  | Mean | -0.019 | -0.037 | -0.001 | **0.036** |
|  | High (+1 SD) | -0.043 | -0.072 | -0.013 | **0.005** |

***Note.*** Significant results (*P* < 0.05) are reported in bold.

| **Table S22: SVR prediction performance for TMT-A from limbic subregional QSM values in the T2DM group in PSM cohort.** | | | | | | | | | | | |
| --- | --- | --- | --- | --- | --- | --- | --- | --- | --- | --- | --- |
| **ROI** | **R²** | **R² 95% CI** | **RMSE** | **RMSE 95% CI** | **MAE** | **MAE 95% CI** | **Spearman’s *r*** | | ***r* 95% CI** | ***P_FDR_*** |  |
| Left cingulate | 0.224 | [0.070, 0.409] | 22.733 | [14.549, 31.091] | 14.206 | [11.196, 17.891] | 0.558 | [0.415, 0.666] | | <0.001 |  |
| Right cingulate | 0.228 | [0.086, 0.371] | 22.671 | [14.330, 22.221] | 14.074 | [11.225, 17.903] | 0.576 | [0.438, 0.693] | | <0.001 |  |
| Left parahippocampal | 0.121 | [-0.242, 0.315] | 24.192 | [16.280, 32.585] | 15.378 | [12.295, 19.02] | 0.496 | [0.349, 0.630] | | <0.001 |  |
| Right parahippocampal | 0.220 | [0.033, 0.450] | 22.794 | [14.193, 22.405] | 13.940 | [11.004, 17.534] | 0.551 | [0.405, 0.676] | | <0.001 |  |
| Left entorhinal | 0.196 | [-0.019, 0.377] | 23.141 | [14.781, 31.459] | 14.459 | [11.437, 17.822] | 0.573 | [0.430, 0.683] | | <0.001 |  |
| Right entorhinal | 0.266 | [0.116, 0.459] | 22.111 | [13.881, 30.961] | 13.509 | [10.744, 16.839] | 0.589 | [0.445, 0.700] | | <0.001 |  |
| ***Note.*** SVR, support vector regression (RBF kernel); nested 5-fold cross-validation with inner-fold grid search. 95% CIs were estimated via 1,000-iteration bootstrap resampling of out-of-sample predictions. | | | | | | | | | | | |

**Table S23: Between-group comparisons of limbic subregional QSM values in the PSM-matched cohort**

**after additional adjustment for DBP, HDL-C, and hypertension.**

| **Region** | **T2DM** | **HC** | **statistic** | ***P* value** | ***P_FDR_*** |
| --- | --- | --- | --- | --- | --- |
| Left cingulate cortex | -2.60 (-9.54, 4.30) | -6.04 (-13.46, 2.68) | 2.795 | **0.035** | 0.069 |
| Right cingulate cortex | -3.83 (-9.60, 3.22) | -5.77 (-12.96, 2.21) | 2.027 | 0.068 | 0.102 |
| Left entorhinal cortex | -15.16 (-35.52, 3.51) | -17.48 (-36.09, 1.09) | 0.052 | 0.84 | 0.996 |
| Right entorhinal cortex | -19.09 (-33.85, -0.07) | -16.18 (-35.64, 0.07) | -0.158 | 0.996 | 0.996 |
| Left parahippocampal | 10.15 (1.76, 17.38) | 8.44 (0.69, 14.41) | 1.945 | **0.016** | **0.049^*^** |
| Right parahippocampal | 14.76 (8.56, 20.75) | 10.72 (5.71, 17.99) | 2.756 | **0.004** | **0.025^*^** |

***Note.*** Significant results (*P* < 0.05) are reported in bold. ^*^ Indicates *P_FDR_* < 0.05
